# KLF15 activates PGC1α to rewire mitochondrial homeostasis and overcome cisplatin resistance in lung adenocarcinoma

**DOI:** 10.1038/s41420-026-03235-3

**Published:** 2026-06-27

**Authors:** Wan Lin, Lijuan Li, Yuwen Bai, Mai Wen, Wenjie Dong, Ruikun Ji, Jianqun Ma, Xiaodong Ling

**Affiliations:** 1 Science and Technology Academic Department of Harbin Medical University Cancer Hospital, No.150 Haping Road, Harbin, 150081 China; 2 Heilongjiang Clinical Research Center for Breast Cancer, Harbin Medical University Cancer Hospital, No.150 Haping Road, Harbin, 150081 China; 3 Department of Thoracic Surgery, Harbin Medical University Cancer Hospital, No.150 Haping Road, Harbin, 150081 China

**Keywords:** Non-small-cell lung cancer, Chemotherapy

## Abstract

Cisplatin resistance represents a major barrier to effective treatment of lung adenocarcinoma (LUAD), yet its metabolic underpinnings remain incompletely defined. Here, we demonstrate that the transcription factor KLF15 governs cisplatin sensitivity by orchestrating mitochondrial biogenesis and redox homeostasis. KLF15 is downregulated in cisplatin-resistant LUAD cells, which display reduced mitochondrial content and suppressed reactive oxygen species (ROS) accumulation. Restoring KLF15 expression resensitizes LUAD cells to cisplatin both in vitro and in vivo. Mechanistically, KLF15 directly transactivates PGC1α, and loss of PGC1α abrogates KLF15-mediated cisplatin sensitization, restoring drug resistance by attenuating apoptosis. Functionally, LUAD subpopulations with low mitochondrial mass or low KLF15 expression exhibit intrinsic resistance, whereas Mito-high xenografts show enhanced therapeutic response. Together, our findings identify a KLF15-PGC1α regulatory axis that dictates mitochondrial reprogramming and cisplatin responsiveness, highlighting a potential therapeutic axis to overcome chemoresistance in LUAD.

## Introduction

Lung adenocarcinoma (LUAD) accounts for nearly 40% of non-small cell lung cancer cases and remains the leading cause of cancer-related death worldwide [1]. Although recent advances in targeted therapies and immune checkpoint inhibitors have extended survival for some patients, platinum-based doublets-most commonly cisplatin combined with pemetrexed-remain a mainstay of first-line treatment for advanced disease [2, 3]. Unfortunately, the clinical efficacy of cisplatin is frequently undermined by the development of intrinsic or acquired resistance, resulting in tumor recurrence, metastasis, and dismal five-year survival rates below 20% [4–7]. A deeper mechanistic understanding of the pathways that govern cisplatin resistance is therefore critical for the design of more durable therapeutic regimens.

Cisplatin exerts its cytotoxic effects primarily through the formation of DNA adducts that block replication and trigger apoptosis [8, 9]. Well-established resistance mechanisms include increased DNA repair capacity (notably via nucleotide excision repair), upregulation of drug efflux transporters (such as MRP2 and CTR1), enhanced detoxification through glutathione and metallothionein, and dysregulation of apoptosis regulators (for instance, BCL-2 family proteins) [10, 11]. However, these paradigms only partially explain the heterogeneous response observed in LUAD patients. Emerging data point to metabolic reprogramming-particularly at the level of mitochondria-as a central contributor to chemoresistance. Mitochondria not only supply ATP via oxidative phosphorylation (OXPHOS) but also regulate reactive oxygen species (ROS) generation and intrinsic apoptotic signaling through cytochrome-c release. Perturbations in mitochondrial mass, dynamics, and bioenergetic function can therefore have profound effects on drug sensitivity and survival under chemotherapeutic stress. Emerging evidence suggests a close association between mitochondrial biogenesis and ROS generation [12]. Increased mitochondrial mass has been observed to elevate ROS levels, particularly under stress conditions [13, 14]. However, it remains unclear whether this biogenesis-driven ROS production contributes to cisplatin resistance in LUAD.

Peroxisome proliferator-activated receptor γ coactivator 1α (PGC1α) is a master regulator of mitochondrial biogenesis and oxidative metabolism [15, 16]. By co-activating nuclear respiratory factors (NRF1/2) and mitochondrial transcription factor A (TFAM), PGC1α orchestrates the transcriptional program required for the assembly of mitochondrial respiratory complexes and the maintenance of mitochondrial DNA. In several solid tumors, including melanoma and breast cancer, elevated PGC1α expression has been linked to increased mitochondrial respiration, antioxidant capacity, and resistance to chemotherapeutic agents [17–19]. Yet in LUAD, the regulatory networks that control PGC1α-and by extension mitochondrial biogenesis-remain poorly defined, and how these pathways intersect with cisplatin sensitivity has not been systematically explored.

Kruppel-like factors (KLFs) are a group of zinc finger-containing transcription factors that play critical roles in modulating gene expression and orchestrating a range of cellular functions [20]. Accumulating research has implicated abnormal KLF activity or expression in the initiation and progression of nearly all forms of human cancers [21]. Among them, KLF15 has been recognized as a key player in various physiological processes as well as tumorigenesis. Altered expression of KLF15 has been associated with the development of several malignancies, such as lung adenocarcinoma, gastric cancer, and breast cancer [22–25]. More recent studies implicate KLF15 in the regulation of cardiac mitochondrial function and in the adaptive response to nutrient stress [26]. However, its functions in tumor cells-and specifically in the context of chemotherapy response-are virtually unexplored. Preliminary expression analyses of LUAD patient cohorts suggest that KLF15 levels are significantly reduced in tumor versus normal lung tissue and correlate with poor prognosis, raising the intriguing possibility that KLF15 may serve as a tumor suppressor whose loss contributes to cisplatin resistance.

In this study, we demonstrate that KLF15 is significantly downregulated at both the mRNA and protein levels in cisplatin-resistant LUAD cell lines, and that reduced KLF15 expression in patient cohorts strongly correlates with shorter overall survival. Through gain- and loss-of-function experiments, we show that KLF15 re-sensitizes resistant cells to cisplatin by directly transactivating the PGC1α promoter, thereby promoting mitochondrial biogenesis, augmenting ROS production, and facilitating apoptosis. Conversely, subpopulations of cells with low mitochondrial content express diminished KLF15 and exhibit marked cisplatin resistance both in vitro and in xenograft models. Collectively, our findings define a novel KLF15-PGC1α-mitochondrial biogenesis axis that dictates cisplatin responsiveness in LUAD and highlight mitochondrial regulatory circuits as promising targets to overcome chemotherapy resistance.

## Results

### KLF15 is downregulated in cisplatin-resistant LUAD cells and its loss correlates with poor clinical outcomes

**Fig. 1:**
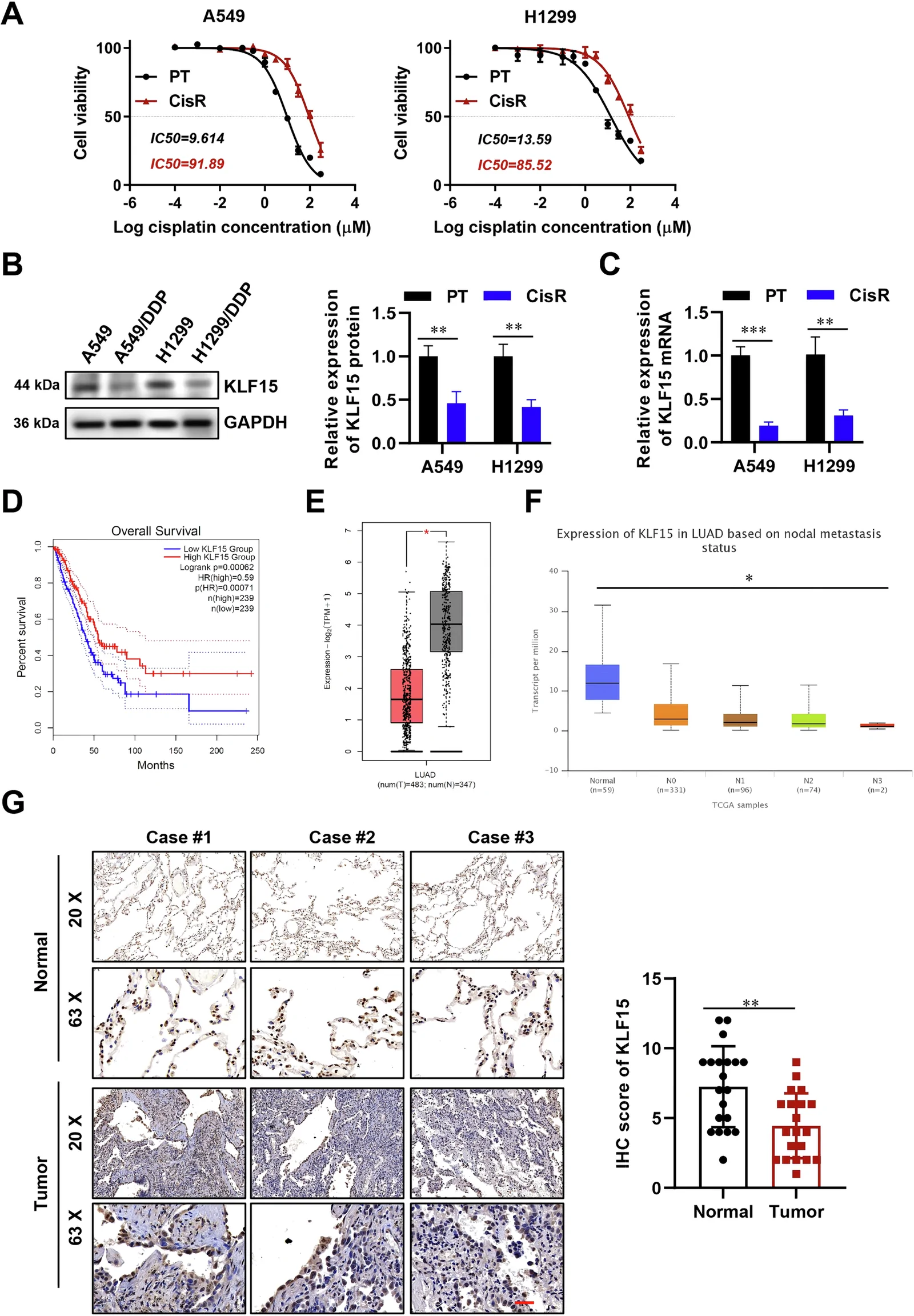
KLF15 is downregulated in cisplatin resistant LUAD cells and correlates with patient prognosis. **A** Cisplatin resistance was established in A549 and H1299 cells by stepwise exposure to increasing concentrations of cisplatin over ~6 months. Resistant and parental cells were treated with a range of cisplatin doses for 48 h, and cell viability was measured by CCK 8 assay. **B** Western blot analysis of endogenous KLF15 expression in cisplatin resistant versus parental LUAD cell lines, showing marked downregulation in resistant cells. **C** qPCR quantification of KLF15 mRNA levels in resistant and parental cell lines, confirming significant reduction in the resistant sublines. **D** Kaplan-Meier overall survival analysis of LUAD patients stratified by KLF15 expression based on the GEPIA database. The data indicates that low KLF15 expression is associated with significantly worse overall survival (HR = 0.59, 95% CI: 0.42 - 0.78, *p* < 0.001). **E** Comparison of KLF15 expression in LUAD tumors versus adjacent normal lung tissues from the GEPIA database, demonstrating significant downregulation in tumor samples. **F** TCGA analysis showing that KLF15 expression is markedly lower in LUAD compared to normal tissue and decreases progressively with advancing nodal metastasis (N stage). **G** Representative immunohistochemical staining for KLF15 in LUAD and matched adjacent normal tissues (*n* = 20). Scale bar, 100 μm. All data are presented as mean ± SD from at least three independent experiments. Statistical significance was determined by the two-tailed unpaired Student’s t-test for **E**, one-way ANOVA followed by Tukey’s post-hoc test for **B**, **C**, and **F**, and the log-rank test for the overall survival analysis in **D**. **P < 0.01, ***P < 0.001.

To investigate the mechanisms driving cisplatin resistance in LUAD, we established resistant sublines (A549/DDP and H1299/DDP) via long-term, stepwise exposure of parental cells to increasing concentrations of cisplatin over approximately six months. The successful induction of resistance was functionally confirmed by significantly elevated IC50 values in the sublines following cisplatin treatment (A549: 91.89 μM vs. 9.61 μM, a 9.56-fold increase; H1299: 85.52 μM vs. 13.59 μM, a 6.29-fold increase) (Fig. 1A). Furthermore, these sublines faithfully recapitulated established clinical resistance mechanisms, exhibiting significant upregulation of MRP2 and ERCC1 alongside a marked downregulation of CTR1 (Fig. S1). Having validated these in vitro models, we next evaluated the expression of the transcription factor KLF15. Consistent with a potential tumor-suppressive role, KLF15 mRNA and protein levels were markedly reduced in both resistant sublines compared to their sensitive parental counterparts (Fig. 1B, C). To determine the clinical relevance of KLF15 suppression, we analyzed publicly available LUAD patient datasets. GEPIA survival analysis indicated that lower KLF15 expression significantly correlates with shorter overall survival in LUAD patients (Fig. 1D). Further analyses using GEPIA and TCGA cohorts revealed that KLF15 is globally downregulated in LUAD tumor tissues compared to normal tissues, and its expression decreases further with advancing nodal metastasis (Fig. 1E, F), indicating that loss of KLF15 is associated with tumor progression. Finally, to validate these in silico findings in vivo, we performed immunohistochemical (IHC) analysis on 20 paired clinical LUAD specimens, confirming that KLF15 protein expression is indeed lower in tumor tissues relative to adjacent normal tissues (Fig. 1G). Collectively, these data demonstrate that KLF15 expression is suppressed in both cisplatin-resistant LUAD cells and clinical tumor specimens, and that its loss is strongly associated with acquired resistance, tumor progression, and adverse patient outcomes.

### Overexpression of KLF15 re-sensitizes resistant LUAD cells to cisplatin in vitro and in vivo

**Fig. 2:**
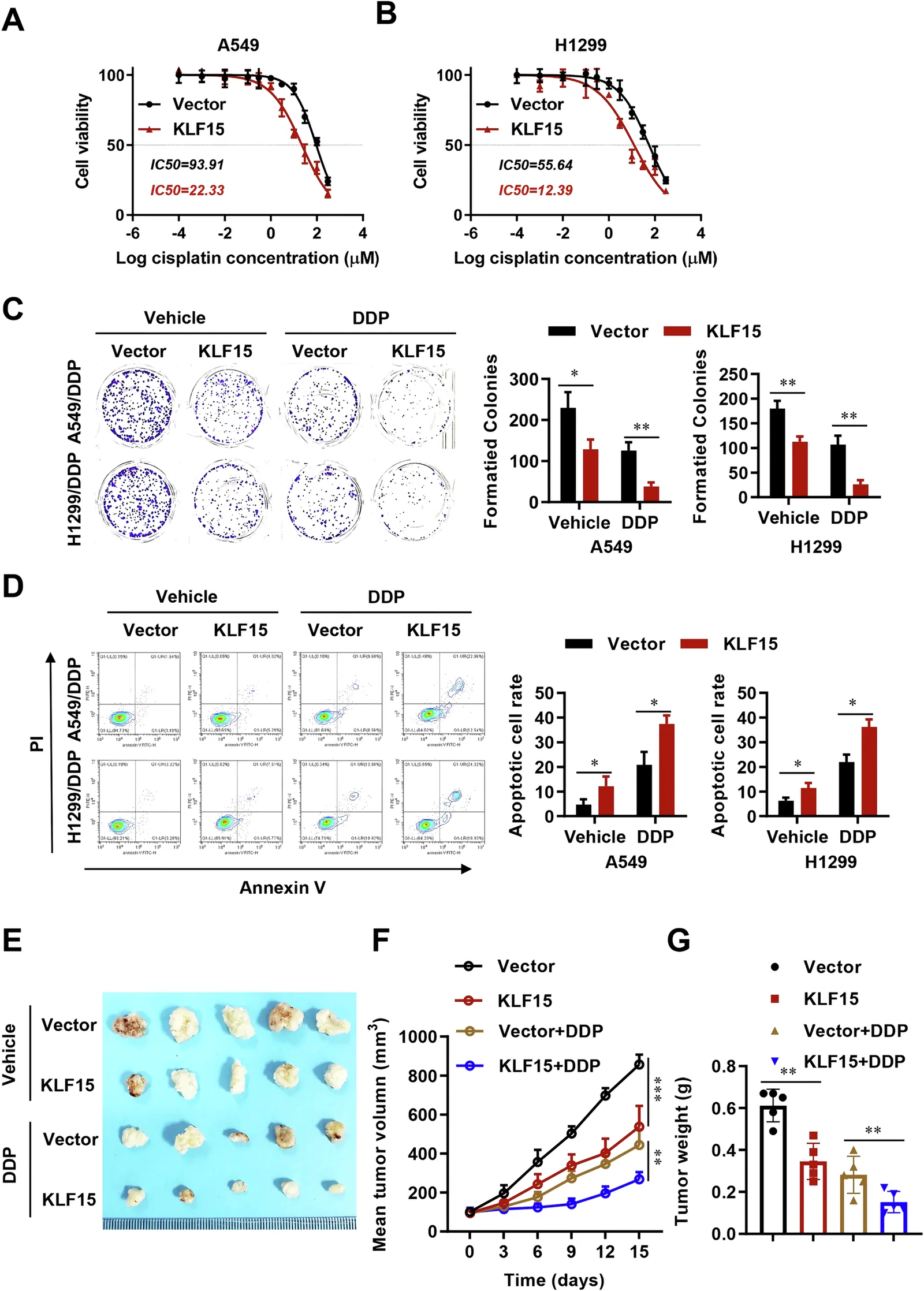
KLF15 overexpression reverses cisplatin resistance in vitro and in vivo. **A**, **B** IC₅₀ determination by CCK 8 assay in vector or KLF15 transfected cisplatin resistant A549 and H1299 cells. **C** Colony formation assays of vector or KLF15 overexpressing resistant LUAD cells treated with vehicle or cisplatin (50 µg/L) showing restored sensitivity upon KLF15 overexpression. **D** Flow cytometric analysis of apoptosis in the same cell populations following cisplatin (50 µg/L) treatment. **E** Nude mice bearing subcutaneous xenografts of vector or KLF15 transfected A549/DDP cells were treated with cisplatin; tumors were photographed 30 days post inoculation. *n* = 5 per group. **F**, **G** Tumor volumes and weights at the end of treatment, showing significant growth inhibition in the KLF15 group. All data are presented as mean ± SEM from at least three independent experiments. Statistical significance for **C**, **D**, **F**, and **G** was determined using one-way ANOVA followed by Tukey’s post-hoc test. *P < 0.05, **P < 0.01, ***P < 0.001.

To assess the functional role of KLF15 in cisplatin resistance, we ectopically expressed KLF15 in A549/DDP and H1299/DDP cells. Overexpression of KLF15 significantly reduced cisplatin IC₅₀ values in both cell lines (Fig. 2A) and restored drug-induced growth inhibition, as shown by reduced colony formation (Fig. 2B) and elevated apoptosis following cisplatin treatment (Fig. 2C). To confirm these findings in vivo, we established subcutaneous xenografts in nude mice using A549/DDP cells with or without KLF15 overexpression, followed by cisplatin administration. Tumor growth was markedly inhibited in the KLF15-overexpressing group (Fig. 2D–F), accompanied by significantly higher apoptosis as detected by TUNEL staining (Fig. S2). These data demonstrate that KLF15 reconstitution reverses cisplatin resistance both in vitro and in vivo.

### Cisplatin-resistant LUAD cells retain mitochondrial function with reduced ROS and mitochondrial content

**Fig. 3:**
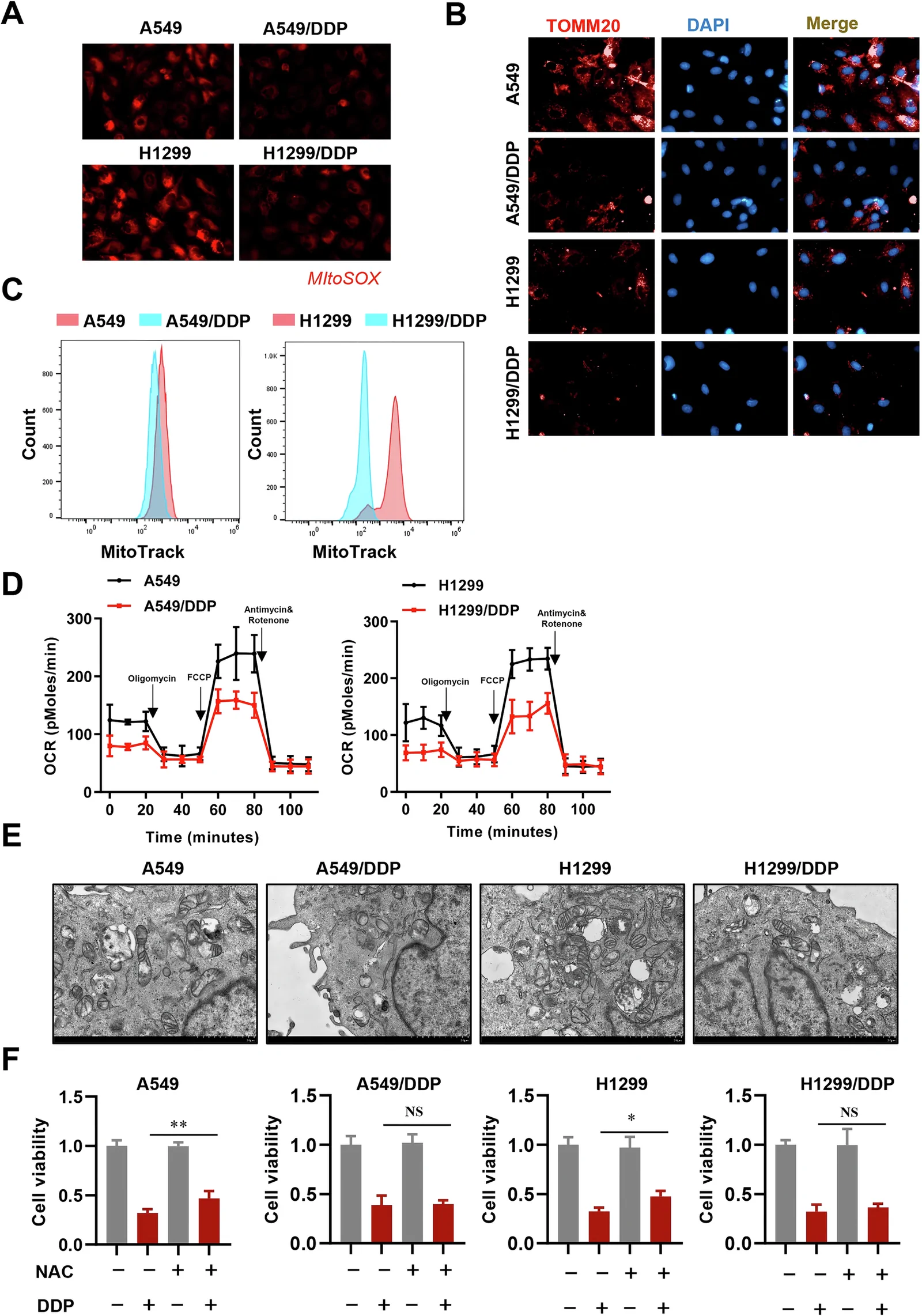
Mitochondrial content, function, and ROS production in cisplatin resistant versus parental LUAD cells. **A** MitoSOX staining and flow cytometry to measure mitochondrial ROS levels. **B** Immunofluorescence staining for TOMM20 to compare mitochondrial mass in resistant and parental cells. **C** Flow cytometric quantification of mitochondrial content using MitoTracker dye. **D** Mitochondrial stress test in a Seahorse XF Analyzer to assess mitochondrial oxygen consumption rate. **E** Transmission electron microscopy of mitochondrial ultrastructure in resistant and parental cells. **F** Cell viability following cisplatin treatment was assessed by CCK-8 assay in the presence or absence of the ROS scavenger N-acetyl-cysteine (NAC). NAC was added to the culture media 3 h prior to cisplatin and was maintained throughout the duration of the drug exposure. All data are presented as mean ± SEM from at least three independent experiments. Statistical significance for F was determined using two-way ANOVA followed by Tukey’s post-hoc test. *P < 0.05, **P < 0.01, ***P < 0.001.

Mitochondrial metabolism has been increasingly implicated in chemoresistance. We observed that cisplatin-resistant LUAD cells displayed significantly lower mitochondrial ROS levels, as assessed by MitoSOX staining (Fig. 3A). Correspondingly, both immunofluorescence staining for TOMM20 and MitoTracker-based flow cytometry revealed decreased mitochondrial content in resistant cells (Fig. 3B, C). Consistently, Seahorse XF analysis demonstrated reduced mitochondrial respiratory activity in resistant cells, including lower basal respiration, maximal respiration, and spare respiratory capacity (Fig. 3D and Fig. S3), indicating a suppression of oxidative phosphorylation despite preserved mitochondrial structure. Transmission electron microscopy (TEM) revealed a reduced number of mitochondria in resistant cells compared to parental cells, although the remaining mitochondria displayed relatively intact ultrastructure with well-defined cristae (Fig. 3E). Notably, across the four experimental groups (Vehicle, Cisplatin, NAC, and Cisplatin + NAC), NAC was added 3 h prior to cisplatin and maintained throughout treatment. Pretreatment with the ROS scavenger N-acetyl-cysteine (NAC) partially rescued cisplatin-induced cell death in parental A549 and H1299 cells. However, it failed to do so in the resistant A549/DDP and H1299/DDP cells, indicating that ROS-mediated apoptosis plays a role in cisplatin sensitivity and is attenuated in the resistant state (Fig. 3F).

### KLF15 regulates mitochondrial biogenesis via transcriptional activation of PGC1α and KLF15-mediated cisplatin sensitivity depends on PGC1α

**Fig. 4:**
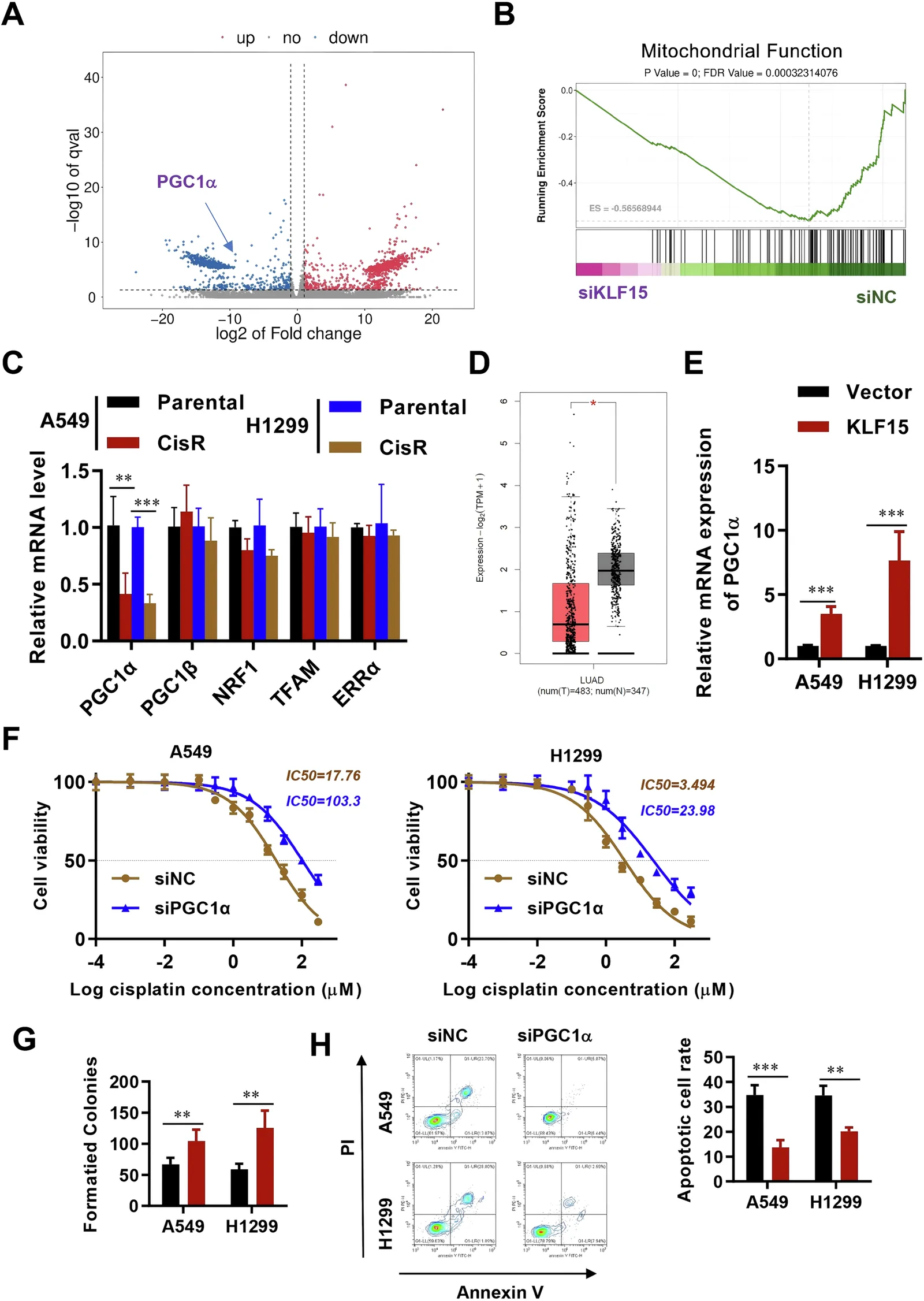
Transcriptomic analysis implicates KLF15 in PGC1α mediated mitochondrial biogenesis. **A** Volcano plot of RNA seq data from A549/DDP cells following KLF15 knockdown, highlighting significant downregulation of PGC1α. **B** Gene set enrichment analysis (GSEA) showing enrichment of mitochondrial function gene sets upon KLF15 depletion. **C** qPCR of mitochondrial biogenesis markers (PGC1α, PGC1β, NRF1, TFAM, and ERRα) in resistant versus parental cells. **D** GEPIA database analysis confirming significant downregulation of PGC1α in LUAD patient samples. **E** Quantitative PCR (qPCR) analysis of *PGC1α* mRNA levels. The data indicate that KLF15 overexpression significantly reverses the upregulation of *PGC1α* in KLF15-overexpressing cells. **F** IC₅₀ determination by CCK 8 assay in in KLF15-overexpressing A549 and H1299 cells with siNC or siPGC1α transfection. **G** Colony formation assays of in KLF15-overexpressing A549 and H1299 cells transfected with siNC or siPGC1α under cisplatin treatment. **H** Representative flow cytometry plots of Annexin V/PI staining showing apoptosis in the indicated groups after cisplatin treatment. All data are presented as mean ± SEM from at least three independent experiments. Statistical significance was determined by the two-tailed unpaired Student’s t-test for **D**, one-way ANOVA followed by Tukey’s post-hoc test for **E**, **G**, and **H**, and the log-rank test for the overall survival analysis in **D**. **P < 0.01, ***P < 0.001.

To dissect the molecular mechanism through which KLF15 modulates mitochondrial dynamics, we performed RNA-sequencing of A549/DDP cells following KLF15 knockdown. To confirm the technical reliability of our sequencing results, several of the most significantly differentially expressed genes were further validated by qPCR. Among the significantly downregulated genes, *PGC1α* (*PPARGC1A*)-a master regulator of mitochondrial biogenesis-emerged as a prominent target (Fig. 4A), and Gene Set Enrichment Analysis (GSEA) revealed a marked enrichment of mitochondrial metabolic gene sets among KLF15-regulated genes (Fig. 4B). Next, to determine whether KLF15 specifically targets *PGC1α* or broadly influences the entire mitochondrial biogenesis program, we systematically evaluated a panel of key regulators within this pathway. Strikingly, qPCR analysis revealed that only *PGC1α* expression was significantly reduced in cisplatin-resistant cells, whereas the mRNA levels of other critical regulators, including *PGC1β*, *NRF1*, *TFAM*, and *ERRα*, remained completely unchanged (Fig. 4C). This confirms the high biological specificity of the KLF15-*PGC1α* regulatory axis. Consistent with these findings, public datasets demonstrated that *PGC1α* is significantly downregulated in LUAD tumors (Fig. 4D). Furthermore, overexpression of KLF15 in both A549 and H1299 cells led to a significant upregulation of *PGC1α* mRNA levels (Fig. 4E), supporting a strong transcriptional regulatory role of KLF15 in promoting *PGC1α* expression.

To determine whether the cisplatin-sensitizing effect of KLF15 is mediated through PGC1α, we performed rescue experiments by silencing PGC1α in KLF15-overexpressing cells. A549 and H1299 cells stably overexpressing KLF15 were transfected with either control siRNA (siNC) or siRNA targeting PGC1α (siPGC1α). Efficient knockdown of PGC1α was confirmed by qRT-PCR and Western blotting (data not shown). Functionally, inhibition of PGC1α significantly reversed the increased cisplatin sensitivity induced by KLF15 overexpression. Specifically, IC50 analysis revealed that PGC1α silencing markedly increased the cisplatin IC50 values in KLF15-overexpressing A549 and H1299 cells (Fig. 4F), indicating a restoration of cisplatin resistance. Consistently, colony formation assays showed that PGC1α knockdown enhanced clonogenic survival of KLF15-overexpressing cells under cisplatin treatment (Fig. 4G). Moreover, Annexin V/PI flow cytometry demonstrated that PGC1α downregulation significantly reduced cisplatin-induced apoptosis in these cells compared with siNC-transfected controls (Fig. 4H). Collectively, these data demonstrate that PGC1α is a critical downstream effector of KLF15 in regulating cisplatin sensitivity, and that loss of PGC1α restores cisplatin resistance in KLF15-overexpressing lung cancer cells. To further validate these findings and rule out potential off-target effects of the siRNA, we employed a pharmacological approach using SR-18292, a specific inhibitor of PGC1α. Strikingly, the addition of SR-18292 closely mirrored the effects of PGC1α genetic ablation. Co-treatment with SR-18292 effectively reversed the cisplatin-sensitizing effect of KLF15, leading to a significant increase in cisplatin IC50 values in KLF15-overexpressing A549 and H1299 cells (Fig. S4A). Furthermore, pharmacological inhibition of PGC1α restored the colony-forming capacity (Fig. S4B) and markedly attenuated cisplatin-induced apoptosis (Fig. S4C) in these cells. Together, these genetic and pharmacological rescue experiments firmly establish that PGC1α is essential for KLF15-mediated cisplatin sensitization.

To verify the functional link between KLF15 and PGC1α suggested by our correlative findings, we performed paired manipulation analyses in A549 and H1299 resistant cells. Cells stably expressing KLF15 or vector control were transiently transfected with siPGC1α or siNC. IC50 assays demonstrated that while KLF15 overexpression significantly sensitized the resistant cells to cisplatin, concurrent silencing of PGC1α effectively reversed this sensitization and restored drug resistance (Fig. S5A). In agreement with these cell viability results, Annexin V/PI flow cytometry showed that the significant increase in drug-induced apoptosis caused by KLF15 was markedly attenuated by PGC1α knockdown (Fig. S5B). Together, these paired analyses confirm that PGC1α is an essential downstream target required for KLF15-mediated reversal of chemoresistance.

### KLF15 directly binds and activates the PGC1α promoter

**Fig. 5:**
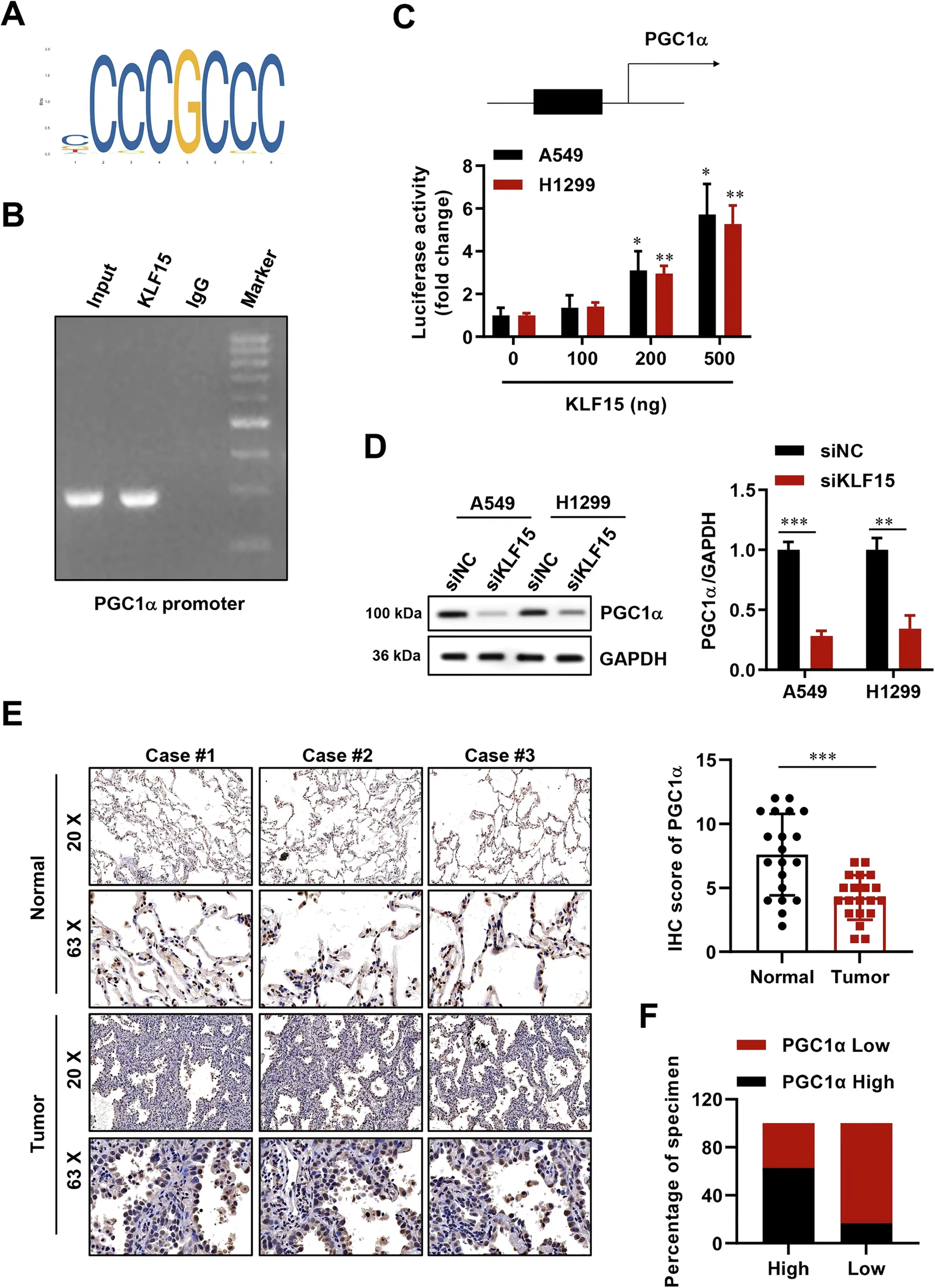
KLF15 directly transactivates the PGC1α promoter. **A** Predicted KLF15 binding sites within the PGC1α promoter region as identified by the hTFtarget database. **B** ChIP PCR validation of KLF15 occupancy at the PGC1α promoter in LUAD cells. **C** Luciferase reporter assays measuring PGC1α promoter activity in A549 and H1299 cells transfected with increasing doses of KLF15 expression plasmid. **D** Western blot showing reduction of PGC1α protein levels following KLF15 knockdown in overexpressing cells. **E** Representative IHC images and quantification of PGC1α expression in LUAD versus adjacent normal tissues (*n* = 20). Scale bar, 100 μm. **F** Distribution of PGC1α staining intensity in specimens stratified by low versus high KLF15 expression. All data are presented as mean ± SEM from at least three independent experiments. Statistical significance was assessed using a two-tailed unpaired Student’s *t*-test for panel **E** and one-way ANOVA with Tukey’s post hoc multiple comparisons test for panels **C**, **D**. *P < 0.05, **P < 0.01, ***P < 0.001.

Using the hTFtarget database, we identified two putative KLF15 binding sites within the proximal promoter region of PGC1α (Fig. 5A). ChIP-PCR confirmed KLF15 occupancy at these loci in LUAD cells (Fig. 5B). Luciferase reporter assays revealed that KLF15 expression dose-dependently enhanced PGC1α promoter activity in both A549 and H1299 cells (Fig. 5C), and KLF15 knockdown significantly decreased PGC1α protein levels (Fig. 5D). IHC staining of clinical LUAD samples showed that PGC1α was reduced in tumors relative to normal tissues (Fig. 5E), and high PGC1α expression correlated positively with KLF15 expression levels (Fig. 5F). These findings establish KLF15 as a direct transcriptional activator of PGC1α in LUAD.

### Low mitochondrial content defines a cisplatin-resistant LUAD subpopulation with reduced KLF15 expression

**Fig. 6:**
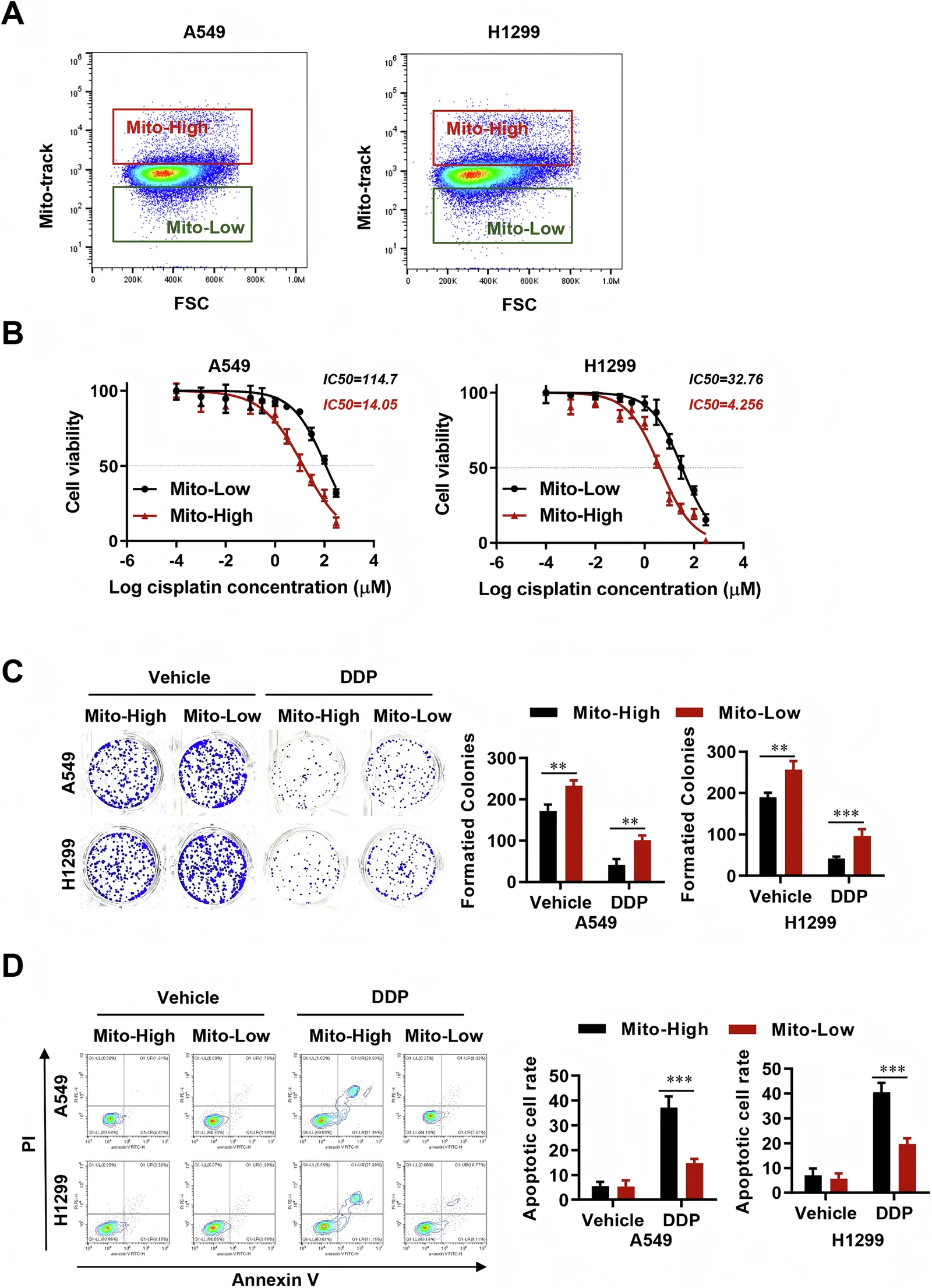
Mitochondrial content predicts cisplatin sensitivity in LUAD cells. **A** FACS sorting of A549 and H1299 cells into Mito high and Mito low populations (top and bottom 10% by MitoTracker fluorescence). **B** CCK 8 assays to determine cisplatin IC₅₀ in Mito high versus Mito low subpopulations. **C** Colony formation assays of Mito high and Mito low cells with or without cisplatin (50 µg/L) treatment. **D** Flow cytometric measurement of early apoptosis in Mito high and Mito low cells following cisplatin treatment. All data are presented as mean ± SEM from at least three independent experiments. Statistical significance for **C**, **D** was determined using two-way ANOVA followed by Tukey’s post-hoc test. **P < 0.01, ***P < 0.001.

LUAD cells such as A549 and H1299 exhibited substantial heterogeneity in mitochondrial content, as revealed by MitoTracker-based flow cytometry analysis. To assess whether this intrinsic variability in mitochondrial mass influences cisplatin sensitivity, cells were labeled with MitoTracker and subjected to fluorescence-activated cell sorting (FACS). The top 10% and bottom 10% of cells, based on mitochondrial staining intensity, were isolated and designated as Mito-high and Mito-low populations, respectively (Fig. 6A). Mito-low cells exhibited significantly higher cisplatin IC₅₀ values than Mito-high cells (Fig. 6B). qPCR and Western blot analysis confirmed that KLF15 mRNA and protein levels were markedly lower in Mito-low cells (Fig. S6). Functionally, Mito-low cells demonstrated enhanced colony formation and resistance to cisplatin-induced apoptosis (Fig. 6C, D), indicating that low mitochondrial content and low KLF15 define a chemoresistant subpopulation.

### Mitochondrial content predicts cisplatin response in vivo

**Fig. 7:**
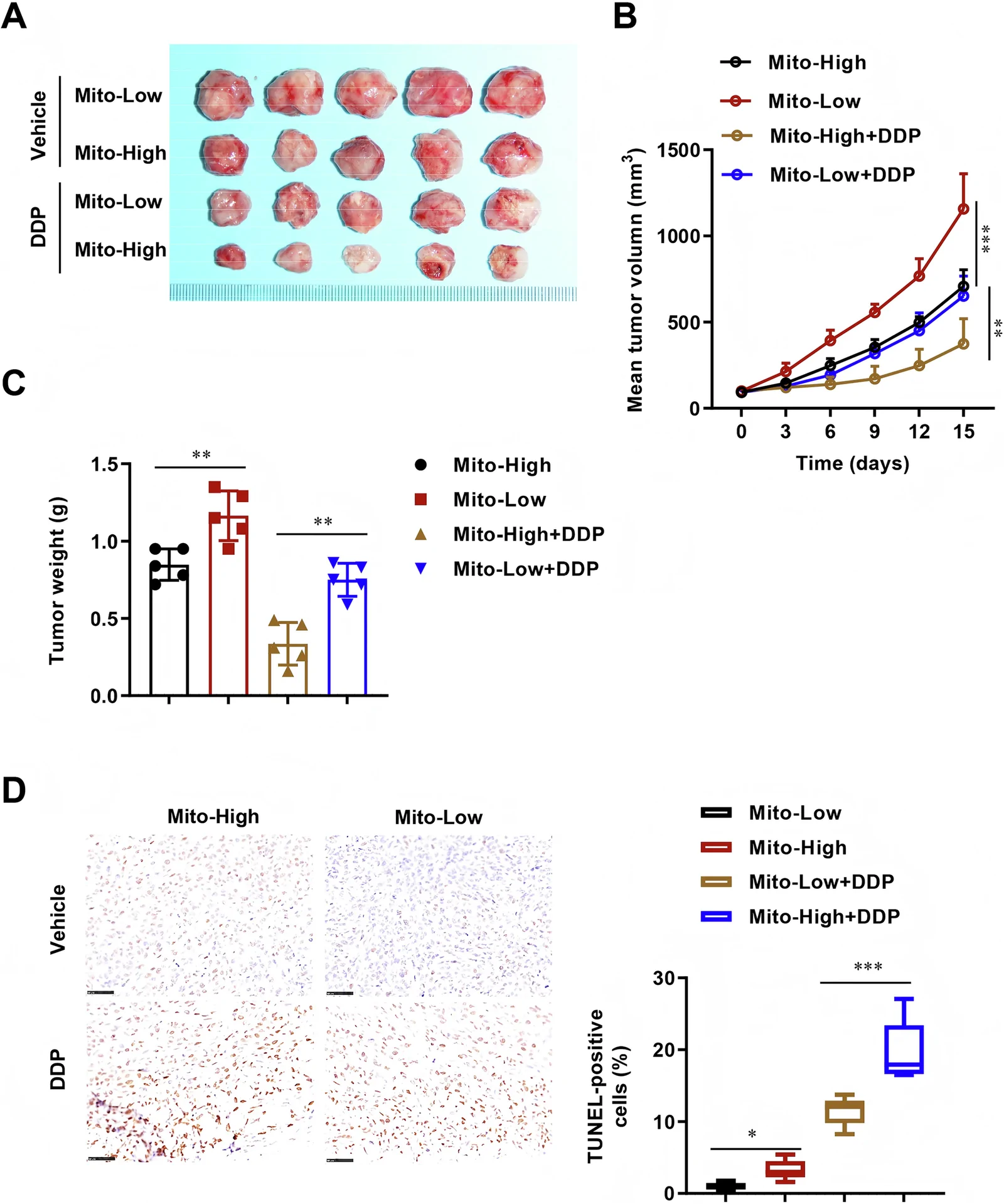
Mitochondrial content governs cisplatin response in vivo. **A** Subcutaneous xenograft of Mito-high and Mito-low A549 cells in nude mice treated with cisplatin; tumor images at day 30 post inoculation. *n* = 5 per group. **B**, **C** Tumor volume and weight measurements at study endpoint. **D** TUNEL staining of xenograft tissues to quantify apoptosis across groups. All data are presented as mean ± SEM from at least three independent experiments. Statistical significance for **B**–**D** was determined using two-way ANOVA followed by Tukey’s post-hoc test. *P < 0.05, **P < 0.01, ***P < 0.001.

**Fig. 8:**
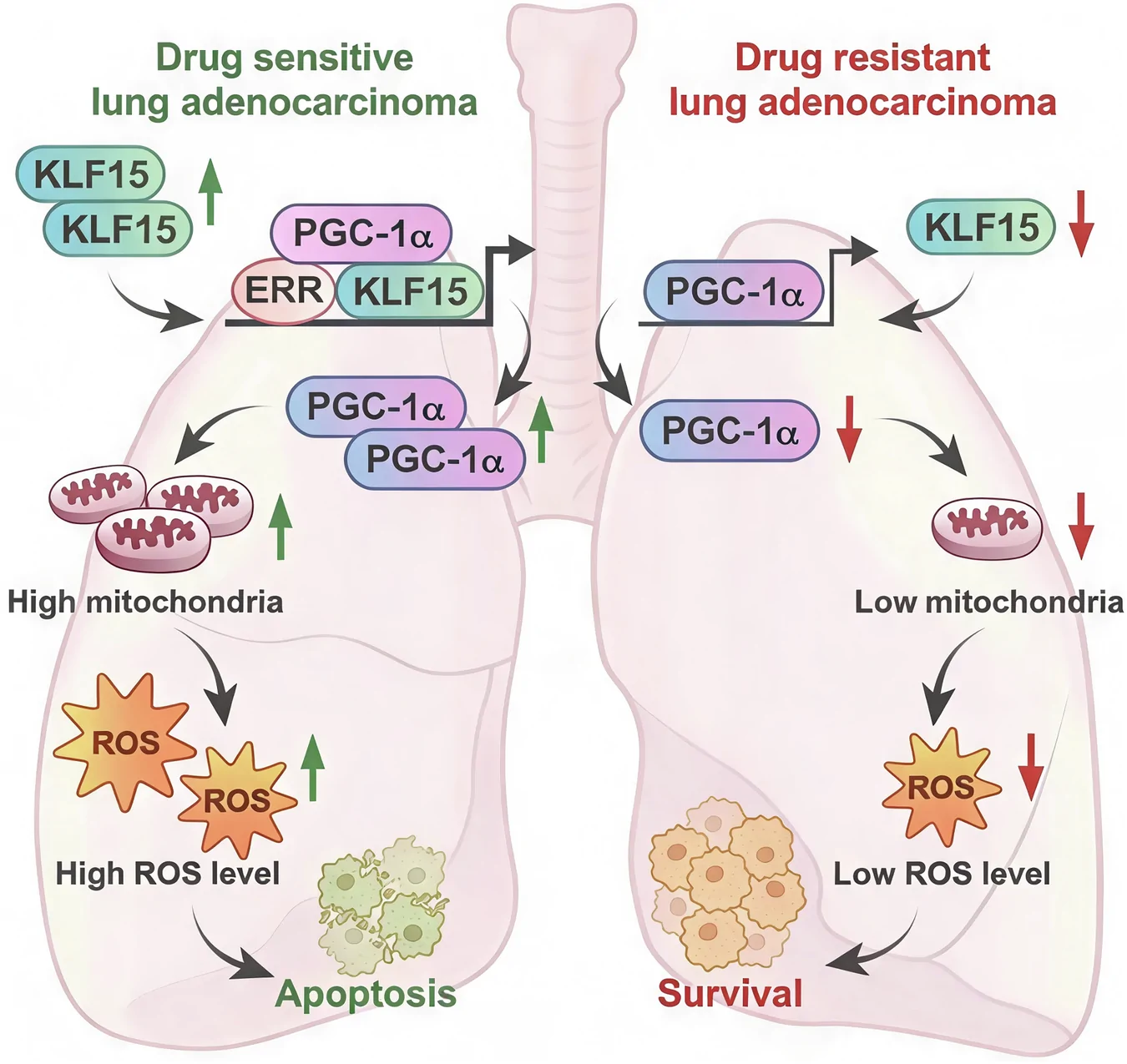
Proposed model. Schematic illustrating that KLF15 transcriptionally activates PGC1α to drive mitochondrial biogenesis, thereby elevating mitochondrial ROS levels, inducing apoptosis, and overcoming cisplatin resistance in LUAD cells.

To extend these findings in vivo, we established xenografts in nude mice using sorted Mito-high and Mito-low A549 cells followed by cisplatin treatment. Mito-high tumors responded significantly better to cisplatin, with reduced tumor volumes and weights compared to Mito-low tumors (Fig. 7A–C). TUNEL staining confirmed increased apoptosis in the Mito-high group (Fig. 7D), supporting a functional link between mitochondrial abundance and chemotherapy responsiveness. Based on these findings, we propose a working model in which KLF15 transcriptionally activates PGC1α, thereby promoting mitochondrial biogenesis and increasing mitochondrial ROS production. This metabolic rewiring sensitizes LUAD cells to cisplatin-induced apoptosis. Loss of KLF15 or mitochondrial content contributes to resistance by dampening ROS-mediated cell death (Fig. 8).

## Discussion

Our study uncovers a previously unrecognized role for KLF15 in modulating chemotherapy response in LUAD by directly engaging the mitochondrial biogenesis program. Although KLF15 has been extensively characterized in normal metabolic tissues, its function in cancer has remained largely unexplored. By demonstrating that KLF15 transactivates the PGC1α promoter to drive mitochondrial mass, respiratory capacity, and ROS generation, we not only identify a mechanistic basis for overcoming cisplatin resistance but also place KLF15 at the nexus of transcriptional and metabolic control in LUAD.

Aberrant expression of KLF15 has been implicated in a variety of diseases [27], and its tumor-suppressive functions have been documented across multiple cancer types. For example, elevated KLF15 levels in breast cancer patients have been associated with improved clinical outcomes. Functional studies revealed that KLF15 overexpression inhibits breast cancer cell proliferation and migration, and induces G0/G1 cell cycle arrest [28]. These findings highlight the therapeutic potential of targeting KLF15 and support its value as a promising tumor biomarker. In LUAD, Wang et al. reported a significant reduction in KLF15 expression; however, the precise role and underlying regulatory mechanisms remain unclear. Our current study further substantiates this observation. Notably, KLF15 expression was markedly reduced in cisplatin-resistant LUAD cells. KLF15 was consistently downregulated in cisplatin-resistant LUAD cells, consistent with its reduced expression in clinical tumor specimens and its association with unfavorable patient prognosis. In early tumor development, loss of KLF15 may favor glycolytic metabolism, reduce oxidative stress, and promote proliferation under hypoxic or nutrient-deprived microenvironments. However, once exposed to repeated cisplatin insult, residual or re-expressed KLF15 appears to confer a survival advantage by bolstering mitochondrial function and detoxification pathways. This adaptive rewiring underscores the plasticity of tumor metabolic networks and suggests that dynamic modulation of KLF15-rather than static expression-dictates therapeutic outcome. The precise signals triggering this KLF15 re-expression during acquired resistance remain to be elucidated, presenting several testable hypotheses for future studies. First, prolonged genotoxic and oxidative stress from repeated cisplatin exposure may activate classical stress-response signaling cascades, which could serve as upstream transcriptional activators of KLF15. Second, acquired resistance is frequently driven by extensive epigenetic remodeling. It is highly plausible that prolonged drug treatment induces alterations in chromatin accessibility, such as DNA demethylation or activating histone modifications at the KLF15 promoter. Additionally, dynamic changes in the non-coding RNA network might alleviate post-transcriptional repression of KLF15 mRNA. Investigating these stress-induced transcriptional and epigenetic mechanisms will be crucial for fully decoding how tumors co-opt KLF15 to navigate chemotherapeutic pressure.

Hypoxia, a hallmark of solid tumors including LUAD, impairs mitochondrial electron transport chain (ETC) function, leading to elevated ROS levels. ROS play dual and context-dependent roles in cancer biology. During early tumorigenesis, moderate ROS levels can promote malignant transformation, while at later stages, excessive ROS accumulation imposes metabolic stress that becomes cytotoxic to cancer cells [29, 30]. Previous studies have shown that cisplatin targets mitochondrial ETC components, triggering ROS production in LUAD cells [31, 32]. To sustain cisplatin resistance and support continued tumor growth, cancer cells appear to adopt adaptive mechanisms that mitigate oxidative damage and escape ROS-induced cell death. Consistent with these findings, we observed increased ROS generation upon cisplatin exposure in parental LUAD cells (data not shown). Interestingly, cisplatin-resistant cells displayed markedly reduced ROS levels following treatment, suggesting an acquired antioxidant capacity that contributes to their resistant phenotype. Mitochondria serve as the primary source of ROS and play a central role in maintaining cellular redox balance. Under hypoxic conditions, mitochondrial function is altered, leading to increased ROS production. A reduction in mitochondrial biogenesis may result in diminished ROS generation, thereby lowering oxidative stress and promoting cellular adaptation to cisplatin exposure. In this study, we observed both decreased mitochondrial biogenesis and attenuated ROS levels in cisplatin-resistant cells, suggesting that metabolic reprogramming of ROS and redox homeostasis is a key adaptive mechanism underlying resistance to cisplatin. Consistent with these observations, our data demonstrate that LUAD cells with elevated mitochondrial content are more susceptible to cisplati, likely due to increased ROS accumulation and enhanced apoptosis. Collectively, these findings highlight mitochondrial biogenesis as a potential predictor of chemotherapy responsiveness, wherein impaired mitochondrial metabolism contributes to LUAD chemoresistance.

PGC1α, a central regulator of mitochondrial biogenesis, displays variable expression patterns across different cancer types and exerts context-dependent effects on tumor progression [33]. Elevated levels of PGC1α have been linked to enhanced metastatic potential in breast cancer, whereas in contrast, reduced metastasis has been observed in melanomas and prostate cancers with high PGC1α expression [34]. Beyond these, its functional significance is increasingly being explored in other malignancies, such as hepatocellular carcinoma, colorectal cancer, renal cell carcinoma, and ovarian cancer [35–38]. In many of these contexts, PGC1α overexpression tends to suppress tumor cell proliferation, while diminished expression often correlates with unfavorable clinical outcomes. However, the specific role of PGC1α in LUAD remains poorly defined, and its therapeutic relevance in this setting is yet to be fully elucidated. Our findings also extend the emerging paradigm that mitochondrial biogenesis constitutes a double-edged sword in cancer. On one hand, enhanced PGC1α-driven OXPHOS can fuel energy-intensive processes such as invasion and metastasis [39]; on the other hand, increased mitochondrial mass sensitizes cells to mitochondria-targeted cytotoxicity via elevated ROS. By channeling KLF15 activity towards mitochondrial expansion, we leverage this vulnerability: resistant cells that maintain low mitochondrial abundance evade cisplatin-induced apoptosis, whereas KLF15 restoration re-sensitizes them. This suggests that combination approaches-pairing cisplatin with agents that activate KLF15 or PGC1α, or directly induce mitochondrial biogenesis- might be potential approaches for increasing chemotherapy sensitivity of LUAD.

In summary, our study defines a KLF15-PGC1α-mitochondrial axis as a critical determinant of cisplatin sensitivity in LUAD. By integrating transcriptional regulation with metabolic control, KLF15 emerges as both a biomarker of drug response and a potential therapeutic target. Future work aimed at pharmacologically modulating KLF15 activity or downstream mitochondrial programs holds promise for overcoming chemotherapy resistance and improving outcomes for patients with lung adenocarcinoma.

## Materials and methods

### Cell culture and establishment of cisplatin‑resistant lines

A549 (ATCC® CCL‑185™) and H1299 (ATCC® CRL‑5803™) human lung adenocarcinoma cells were obtained from the American Type Culture Collection (ATCC, Manassas, VA, USA). Cells were maintained in RPMI‑1640 medium (Cat. #11875‑093, Thermo Fisher Scientific, Waltham, MA, USA) supplemented with 10% fetal bovine serum (FBS; Cat. #04‑001‑1 A, Biological Industries, Beit Haemek, Israel) and 1% penicillin–streptomycin (Cat. #15140‑122, Thermo Fisher Scientific, USA) at 37 °C in a humidified atmosphere containing 5% CO₂. To generate cisplatin‑resistant sublines (A549/DDP and H1299/DDP), parental cells were exposed to stepwise increasing concentrations of cisplatin (starting at 0.5 µg/mL up to 5 µg/mL; Cat. #P4394, Sigma‑Aldrich, St. Louis, MO, USA) over ~6 months. Resistance was confirmed by determining the half‑maximal inhibitory concentration (IC₅₀) via CCK‑8 assay.

### Cell viability (CCK‑8) assay

Cells (5 × 10³/well) were seeded in 96‑well plates and, after overnight attachment, treated with graded concentrations of cisplatin for 48 h. CCK‑8 reagent (10 µL) was added per well and incubated for 2 h at 37 °C. Absorbance was measured at 450 nm using a SpectraMax iD5 microplate reader (Molecular Devices, San Jose, CA, USA). IC₅₀ values were calculated using GraphPad Prism 9.0 (GraphPad Software, San Diego, CA, USA).

### Colony formation assay

Cells (1 × 10³/well) were plated in six‑well plates and cultured for 24 h before treatment with cisplatin (50 µg/L) or vehicle. After 10 days, colonies were fixed with 4% paraformaldehyde (Cat. #P6148, Sigma‑Aldrich, USA) for 20 min, stained with 0.5% crystal violet (Cat. #C0775, Sigma‑Aldrich, USA) for 30 min, and counted manually.

### Mitochondrial mass assessment

To assess mitochondrial content in LUAD cells, cells were incubated with 100 nmol/L MitoTracker Red CMXRos (Cell Signaling Technology, #9082) in complete culture medium for 30 min at 37 °C. Following staining, cells were washed twice with PBS and subjected to flow cytometric analysis.

### Apoptosis detection

Apoptosis was evaluated using the Annexin V-FITC/PI Apoptosis Detection Kit (Vazyme) according to the manufacturer’s protocol. For TUNEL staining, tumor tissues from animal models were sectioned at a thickness of 4 μm. Apoptotic cells were identified and quantified using the TUNEL BrightGreen Apoptosis Detection Kit (Vazyme) following the supplied instructions.

### Western blotting

Cells were lysed in RIPA buffer (Cat. #89900, Thermo Fisher Scientific, USA) supplemented with protease and phosphatase inhibitors (Cat. #78440, Thermo Fisher Scientific, USA). Protein concentrations were determined by BCA assay (Cat. #23227, Thermo Fisher Scientific, USA). Equal amounts (20 µg) were separated by SDS–PAGE (10% gel), transferred to PVDF membranes (Cat. #IPVH00010, Millipore, Burlington, MA, USA), blocked with 5% non‑fat milk in TBST for 1 h, and incubated overnight at 4 °C with primary antibodies (1:1,000). After washing, membranes were incubated with HRP‑conjugated secondary antibodies (1:5,000) for 1 h at room temperature. Bands were visualized using ECL substrate (Cat. #32106, Thermo Fisher Scientific, USA) and imaged on a ChemiDoc MP system (Bio‑Rad, Hercules, CA, USA).

### Quantitative real‑time PCR (qPCR)

Total RNA was extracted using TRIzol™ Reagent (Cat. #15596026, Thermo Fisher Scientific, USA), and 1 µg was reverse‑transcribed with the PrimeScript™ RT Reagent Kit (Cat. #RR047A, Takara Bio, Kusatsu, Japan). qPCR was performed using SYBR® Premix Ex Taq™ II (Cat. #RR820A, Takara Bio, Japan) on a QuantStudio™ 6 Flex Real‑Time PCR System (Applied Biosystems, Foster City, CA, USA). Primer sequences are listed in Supplementary Table S1. Relative expression was calculated by the 2^⁻ΔΔCt^ method using GAPDH as internal control.

### RNA‑seq and gene set enrichment analysis (GSEA)

A549/DDP cells were transfected with siKLF15 or control siRNA and total RNA was subjected to paired‑end sequencing on an Illumina NovaSeq 6000 (Illumina, San Diego, CA, USA) by Novogene (Beijing, China). Differential expression was analyzed with DESeq2, and GSEA was performed using the GSEA software v4.1.0 (Broad Institute, Cambridge, MA, USA) against the “Hallmark” and “Reactome” gene sets.

### Chromatin immunoprecipitation (ChIP)-PCR

ChIP was conducted using the MAGnify™ Chromatin Immunoprecipitation System (Cat. #49‑2024, Thermo Fisher Scientific, USA). Briefly, cells were crosslinked with 1% formaldehyde, lysed, and chromatin sheared by sonication to ∼200–500 bp fragments. Immunoprecipitation was performed with anti‑KLF15 or IgG control (Cat. #12‑370, Millipore, USA). Purified DNA was analyzed by PCR using primers flanking the predicted KLF15 binding sites in the PGC1α promoter (Supplementary Table S1).

### Luciferase reporter assay

Wild‑type PGC1α promoter (–1 kb to +200 bp) was cloned into the pGL3‑Basic vector (Promega, Madison, WI, USA). A549 and H1299 cells were co‑transfected with pGL3‑PGC1α promoter, pRL‑TK Renilla control (Promega, USA), and increasing amounts of KLF15 expression plasmid using Lipofectamine™ 3000 (Cat. #L3000015, Thermo Fisher Scientific, USA). After 48 h, luciferase activity was measured with the Dual‑Luciferase® Reporter Assay System (Cat. #E1910, Promega, USA) on a GloMax® Navigator luminometer (Promega, USA).

### Immunohistochemistry (IHC)

Formalin‑fixed paraffin‑embedded LUAD tissue sections (*n* = 20 pairs) were deparaffinized, rehydrated, and subjected to antigen retrieval in citrate buffer (pH 6.0). Sections were blocked in 5% BSA and incubated overnight at 4 °C with primary antibodies against KLF15 (1:200) or PGC1α (1:150). After incubation with biotinylated secondary antibody (Cat. #PK‑4002, Vector Laboratories, Burlingame, CA, USA) and ABC reagent (Cat. #PK‑4000, Vector Laboratories, USA), signals were developed using DAB substrate (Cat. #SK‑4100, Vector Laboratories, USA). Images were captured on an Olympus BX53 microscope (Olympus, Tokyo, Japan).

### Seahorse XF mitochondrial stress test

Cells were seeded at 2 × 10⁴/well in XF96 plates (Cat. #101085‑004, Agilent Technologies, USA) and incubated overnight. The assay was performed according to manufacturer’s protocol, sequentially injecting oligomycin (1 µM), FCCP (1 µM), and rotenone/antimycin A (0.5 µM each). Oxygen consumption rates (OCR) were recorded on a Seahorse XF96 Analyzer (Agilent Technologies, USA) and analyzed with Wave software.

### Transmission electron microscopy (TEM)

Cells were fixed in 2.5% glutaraldehyde (Cat. #16210, Electron Microscopy Sciences, Hatfield, PA, USA) for 2 h, post‑fixed in 1% osmium tetroxide (Cat. #19110, Electron Microscopy Sciences, USA), dehydrated through graded ethanol, and embedded in Epon resin. Ultrathin sections (70 nm) were stained with uranyl acetate and lead citrate, and imaged using a JEOL JEM‑1230 transmission electron microscope (JEOL Ltd., Tokyo, Japan).

### In vivo xenograft studies

All animal protocols were approved by the Institutional Animal Care and Use Committee of Harbin Medical University Cancer Hospital. Female BALB/c nude mice (4–6 weeks old; Shanghai SLAC Laboratory Animal Co., Ltd., China) were randomly assigned to experimental groups (*n* = 5 per group). Mice were subcutaneously injected in the flank with 5 × 10⁶ A549/DDP cells stably expressing vector or KLF15 (for Fig. 2) or sorted Mito‑high/Mito‑low A549 cells (for Fig. 7) in 100 µL PBS: Matrigel (1: 1; Cat. #356237, Corning, Corning, NY, USA). When tumors reached ~100 mm³, mice received intraperitoneal injections of cisplatin (5 mg/kg) twice weekly for 3 weeks. Tumor dimensions were measured with calipers, and volume was calculated as (length × width²)/2. At study endpoint, tumors were excised, weighed, and processed for histology.

### TUNEL assay

Apoptotic cells in xenograft tissue sections were detected using the In Situ Cell Death Detection Kit, TMR Red (Cat. #12156792910, Roche Diagnostics, Mannheim, Germany) according to manufacturer’s instructions. Sections were counterstained with DAPI (Cat. #D9542, Sigma‑Aldrich, USA) and imaged by fluorescence microscopy (Olympus BX53).

### Statistical analysis

Data are presented as mean ± SD from at least three independent experiments. Statistical comparisons were performed using two‑tailed Student’s t‑test or ANOVA with Tukey’s post hoc test in GraphPad Prism 9.0. Survival curves were plotted by the Kaplan–Meier method and compared by log‑rank test. *p* < 0.05 was considered statistically significant.

## Supplementary information

Supplementary Figure Legends

Supplementary Figure

Table S1

full length uncropped original western blots

## Data Availability

Data will be made available on request.
