# Supplementary material for: KLF15 activates PGC1α to rewire mitochondrial homeostasis and overcome cisplatin resistance in lung adenocarcinoma
Source: Cell Death Discov. 2026 Jun 27;12:369. doi: 10.1038/s41420-026-03235-3 (PMC13572412; doi:10.1038/s41420-026-03235-3)
Supplement: Supplementary file 1 [file 41420_2026_3235_MOESM2_ESM.pdf]

Figure S1

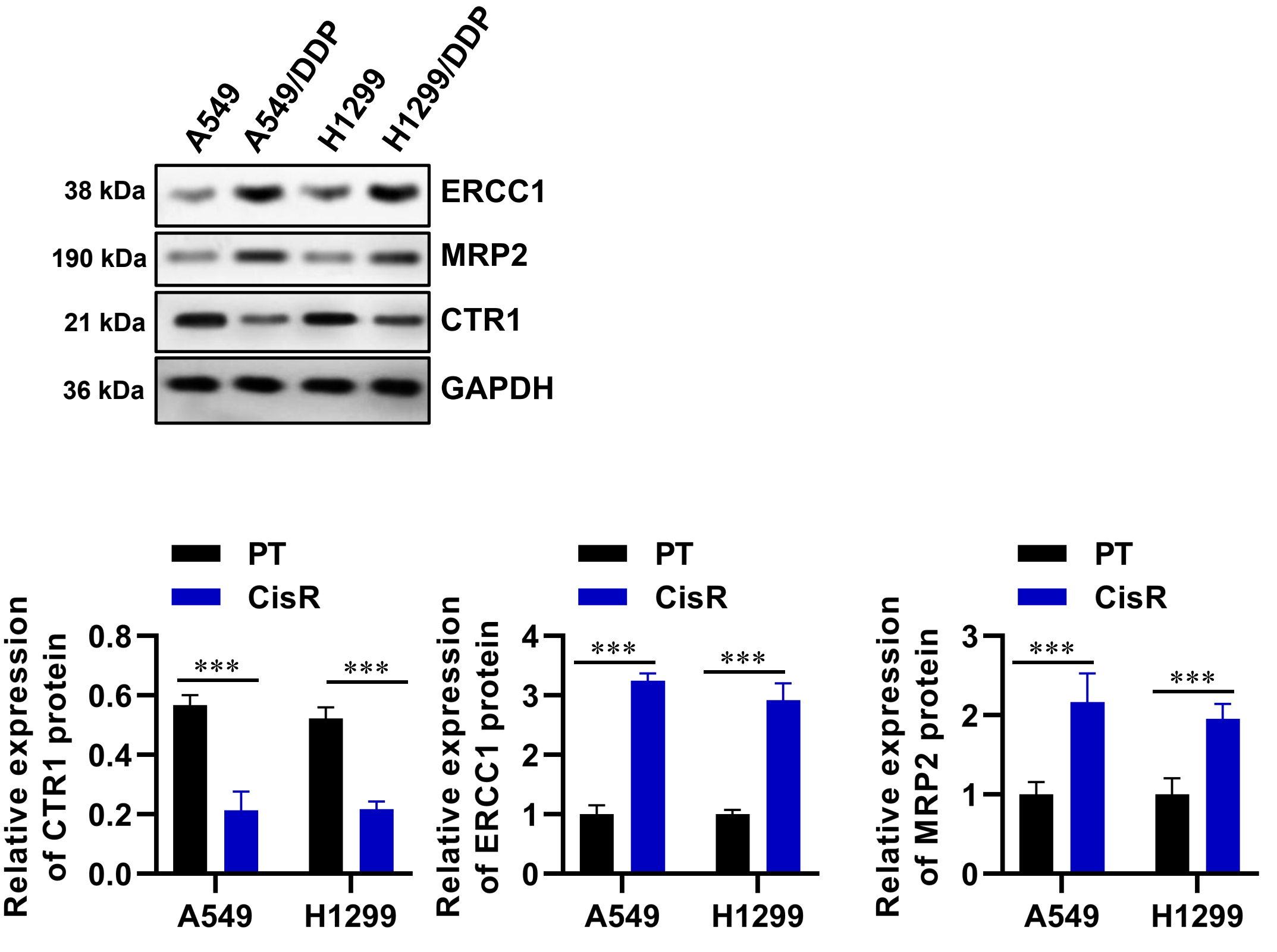

A549
A549/DDP
H1299
H1299/DDP
38 kDa
ERCC1
190 kDa
MRP2
21 kDa
CTR1
36 kDa
GAPDH
PT
CisR
Relative expression of CTR1 protein
0.0
0.2
0.4
0.6
0.8
***
***
A549
H1299
PT
CisR
Relative expression of ERCC1 protein
0
1
2
3
4
***
***
A549
H1299
PT
CisR
Relative expression of MRP2 protein
0
1
2
3
***
***
A549
H1299

Figure S2

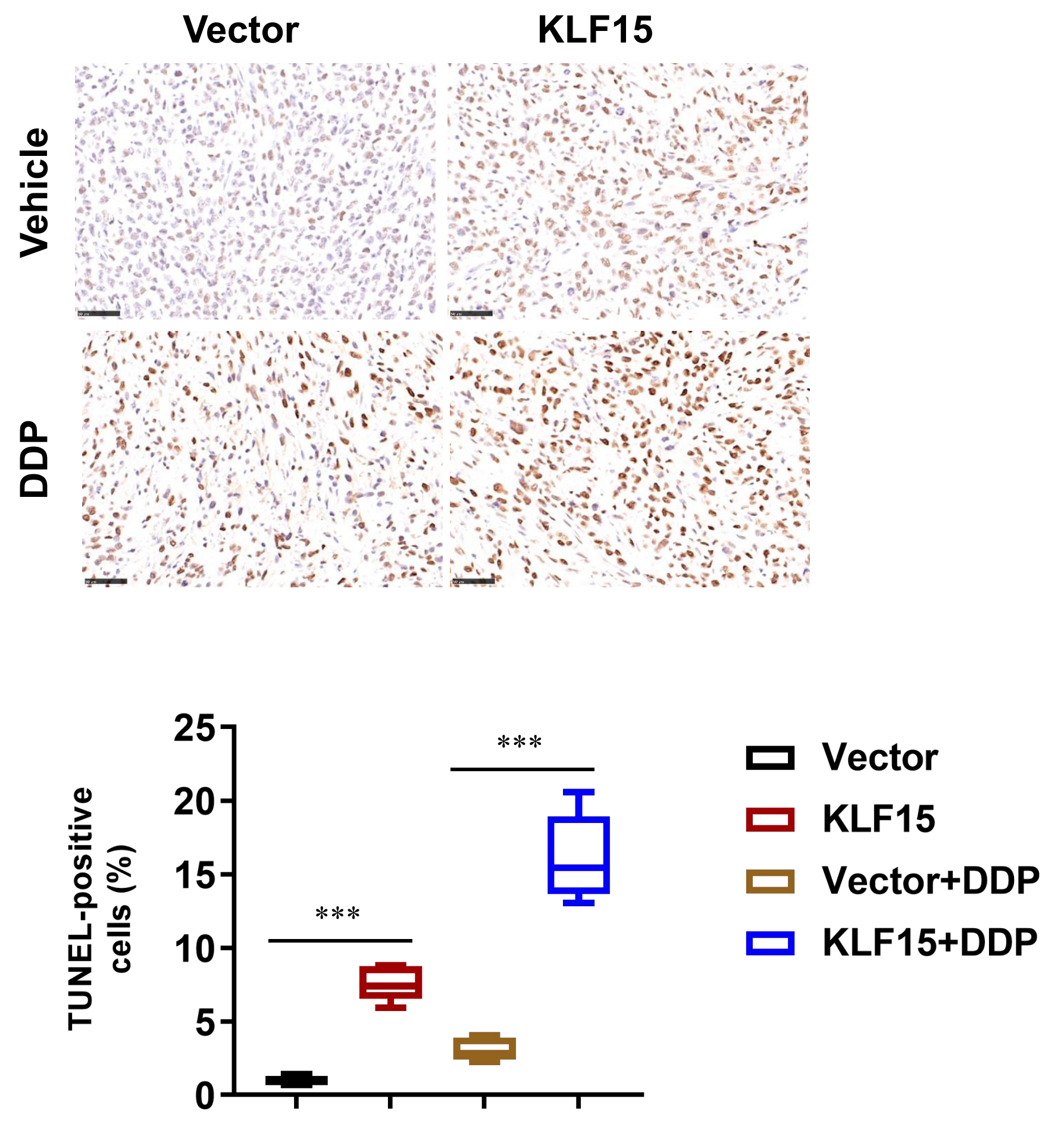

Vector
KLF15
Vehicle
DDP
TUNEL-positive cells (%)
25
20
15
10
5
0
***
***
Vector
KLF15
Vector+DDP
KLF15+DDP

Figure S3

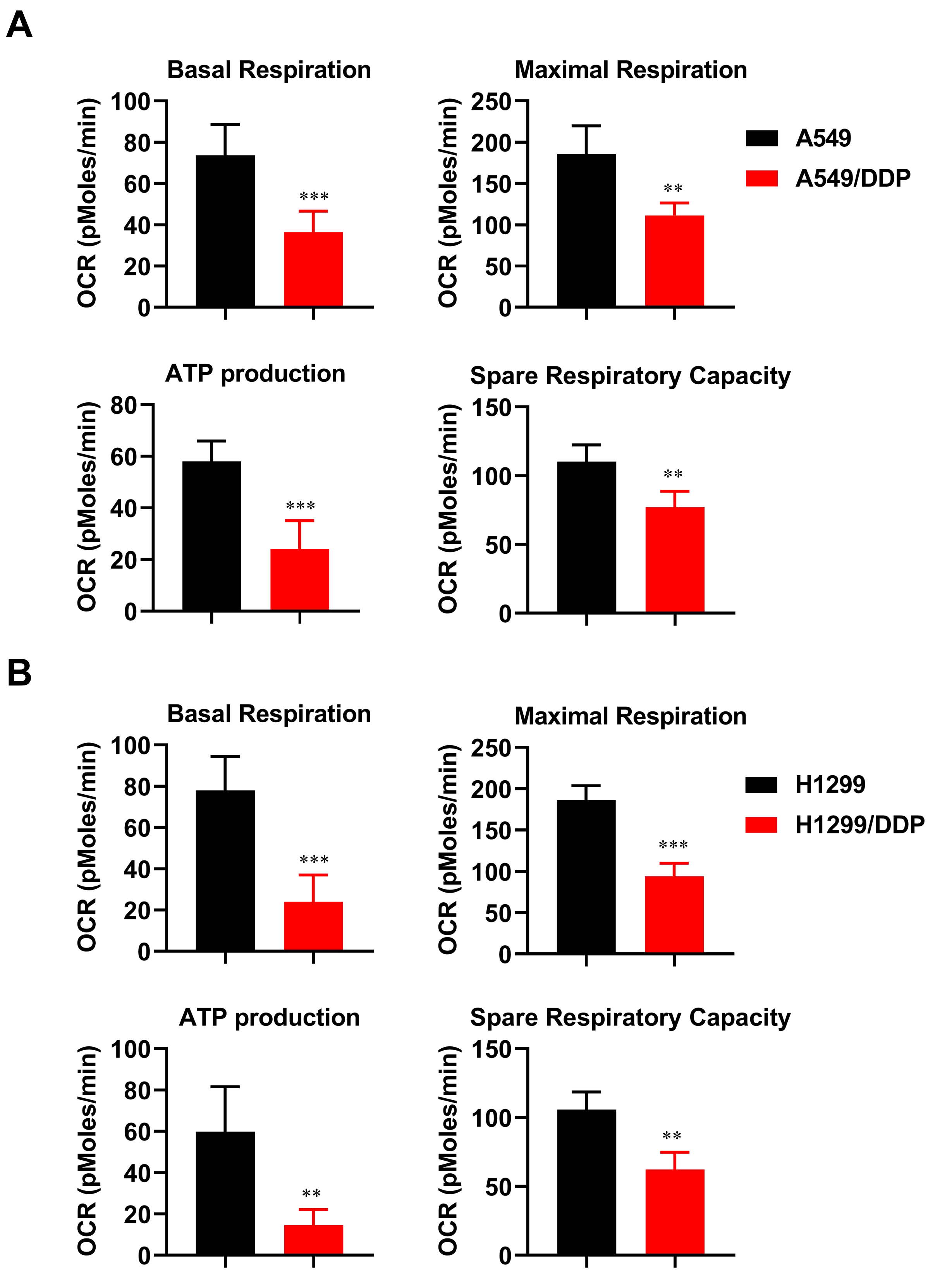
A
Basal Respiration
Maximal Respiration
ATP production
Spare Respiratory Capacity
OCR (pmoles/min)
A549
A549/DDP
***
**
B
Basal Respiration
Maximal Respiration
ATP production
Spare Respiratory Capacity
OCR (pmoles/min)
H1299
H1299/DDP
***
**

Figure S4

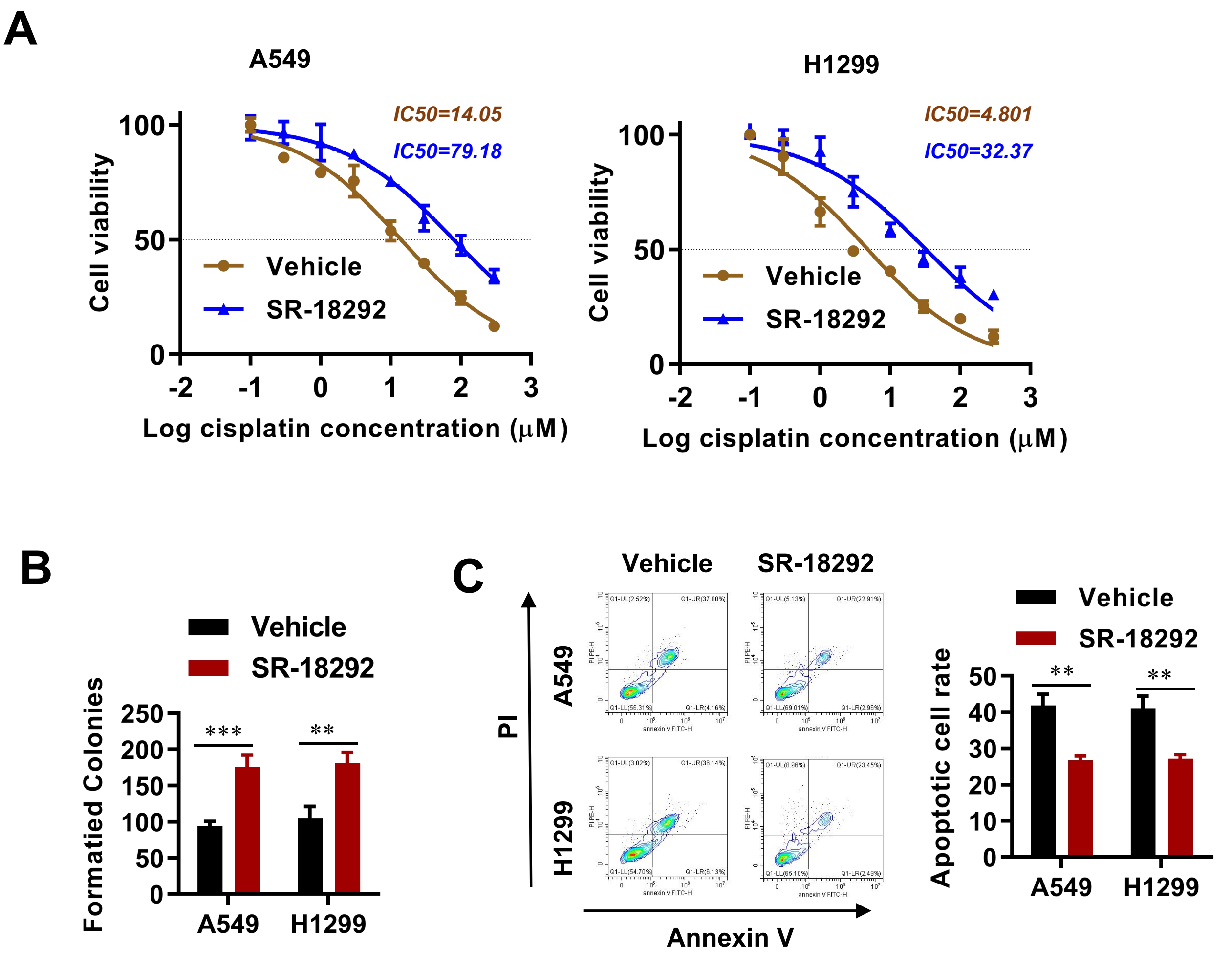
A
A549
H1299
IC50=14.05
IC50=79.18
IC50=4.801
IC50=32.37
Cell viability
Vehicle
SR-18292
Log cisplatin concentration (μM)
B
Formatied Colonies
A549
H1299
C
Vehicle
SR-18292
PI
Annexin V
Apoptotic cell rate

Figure S5

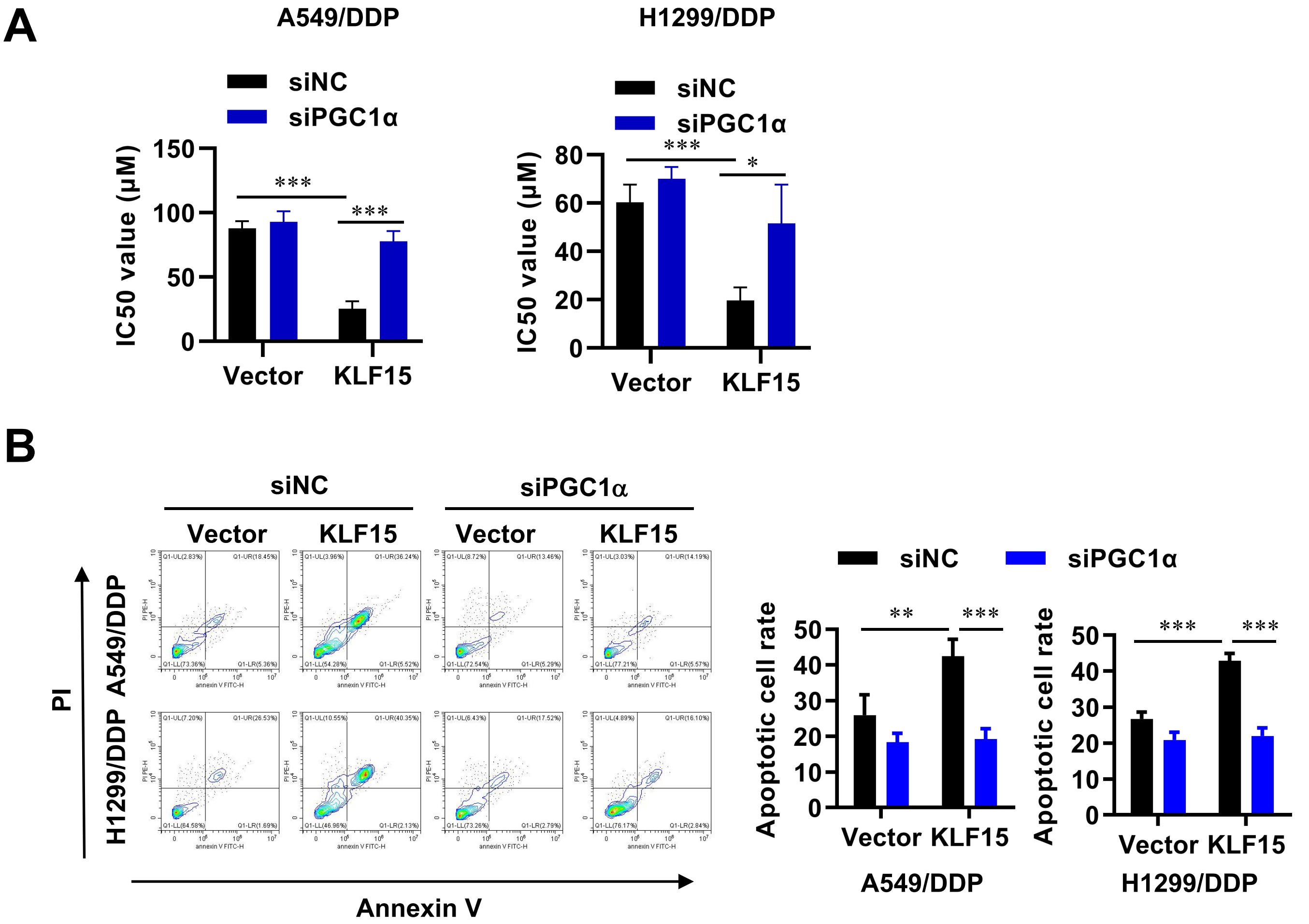
A
A549/DDP
H1299/DDP
siNC
siPGC1α
IC50 value (μM)
Vector
KLF15
***
*
B
siNC
siPGC1α
Vector
KLF15
PI
A549/DDP
H1299/DDP
Annexin V
Apoptotic cell rate
**
***

Figure S6

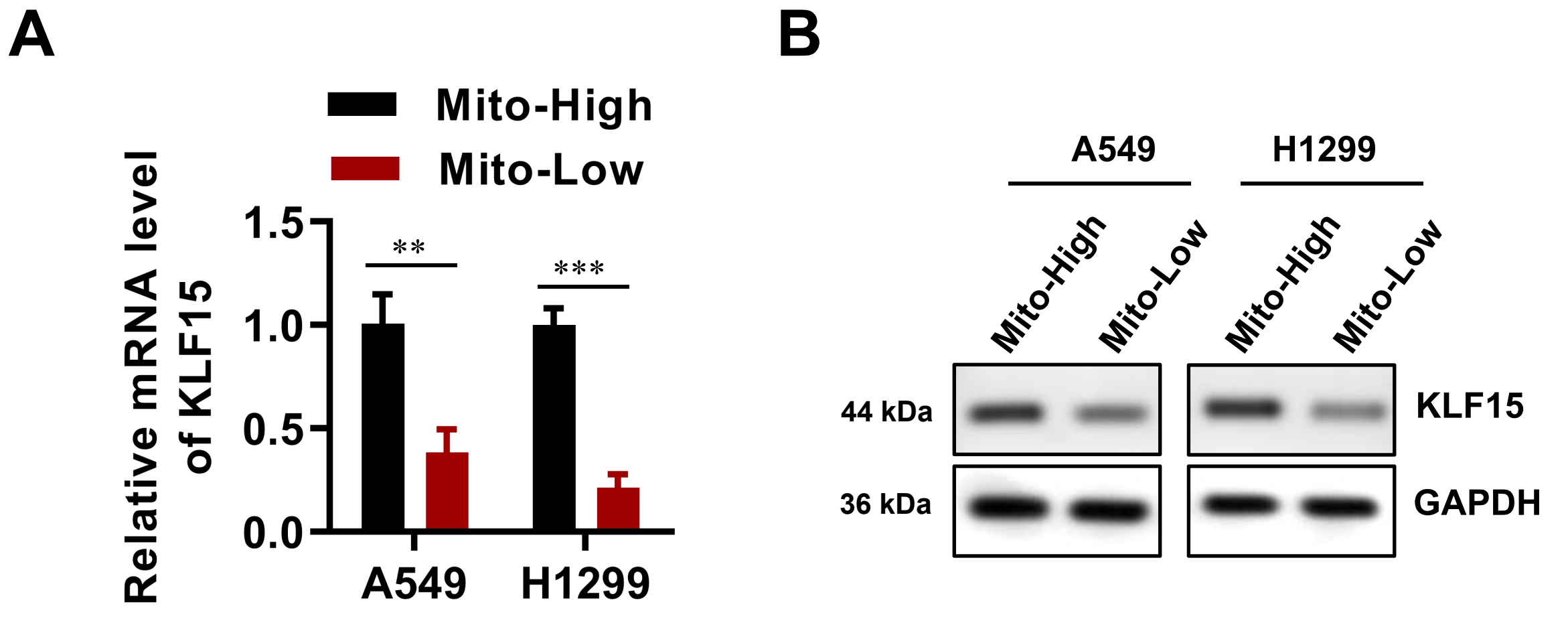
A
Mito-High
Mito-Low
Relative mRNA level of KLF15
1.5
1.0
0.5
0.0
**
***
A549
H1299
B
A549
H1299
Mito-High
Mito-Low
Mito-High
Mito-Low
44 kDa
36 kDa
KLF15
GAPDH
