# Supplementary material for: KLF15 activates PGC1α to rewire mitochondrial homeostasis and overcome cisplatin resistance in lung adenocarcinoma
Source: Cell Death Discov. 2026 Jun 27;12:369. doi: 10.1038/s41420-026-03235-3 (PMC13572412; doi:10.1038/s41420-026-03235-3)
Supplement: Supplementary file 2 [file 41420_2026_3235_MOESM4_ESM.pdf]

Figure 1B

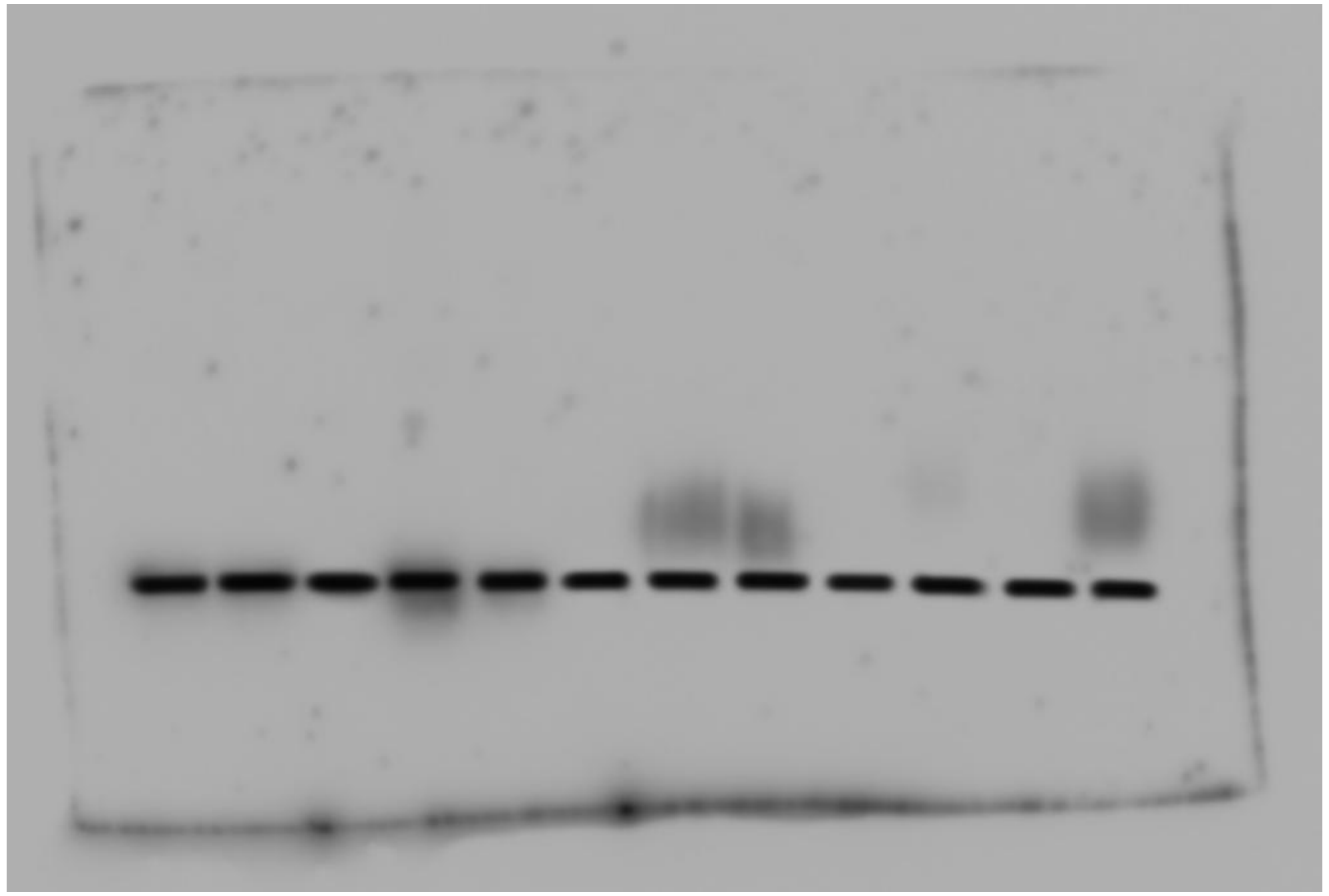

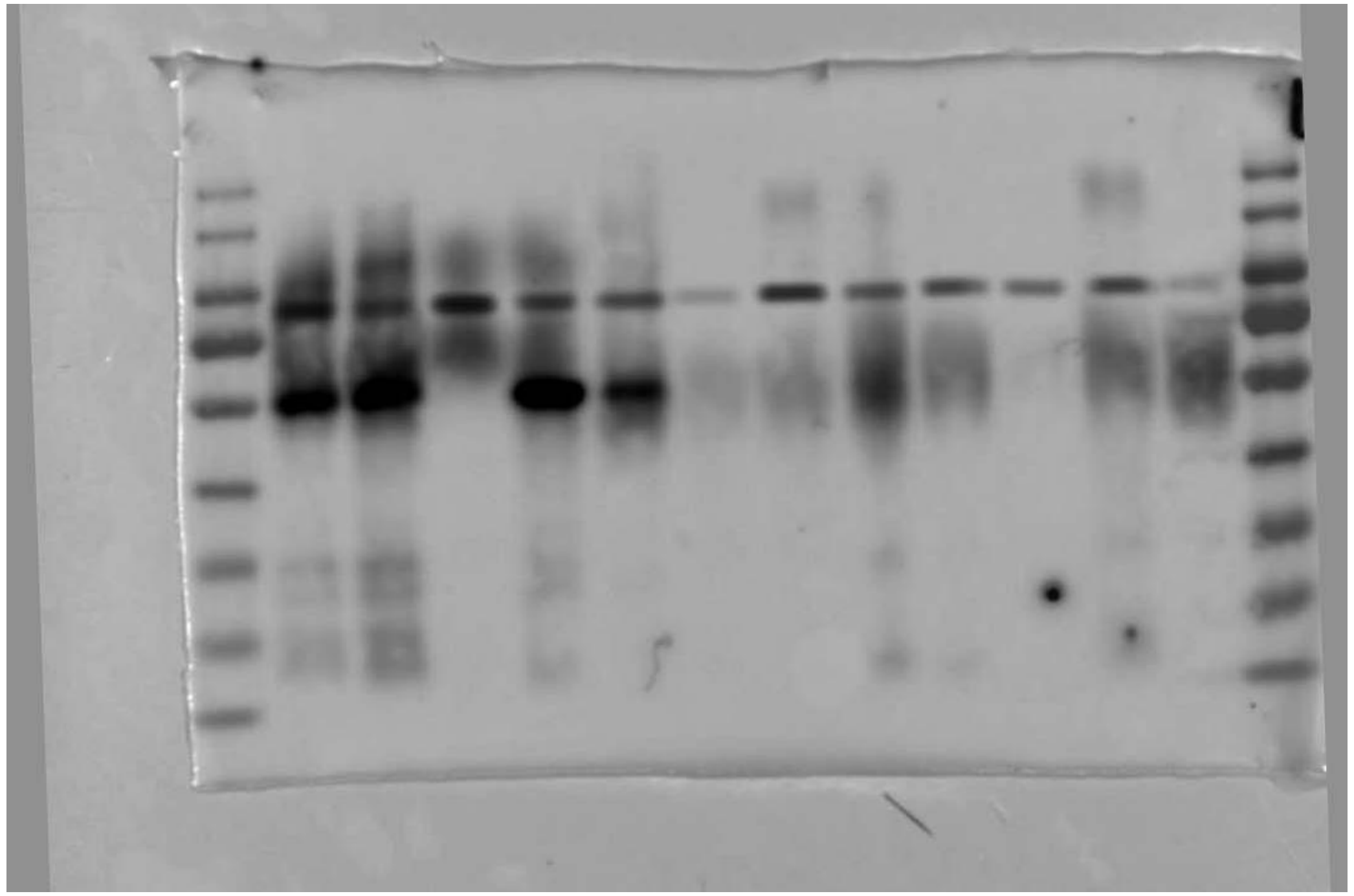

Figure 5D

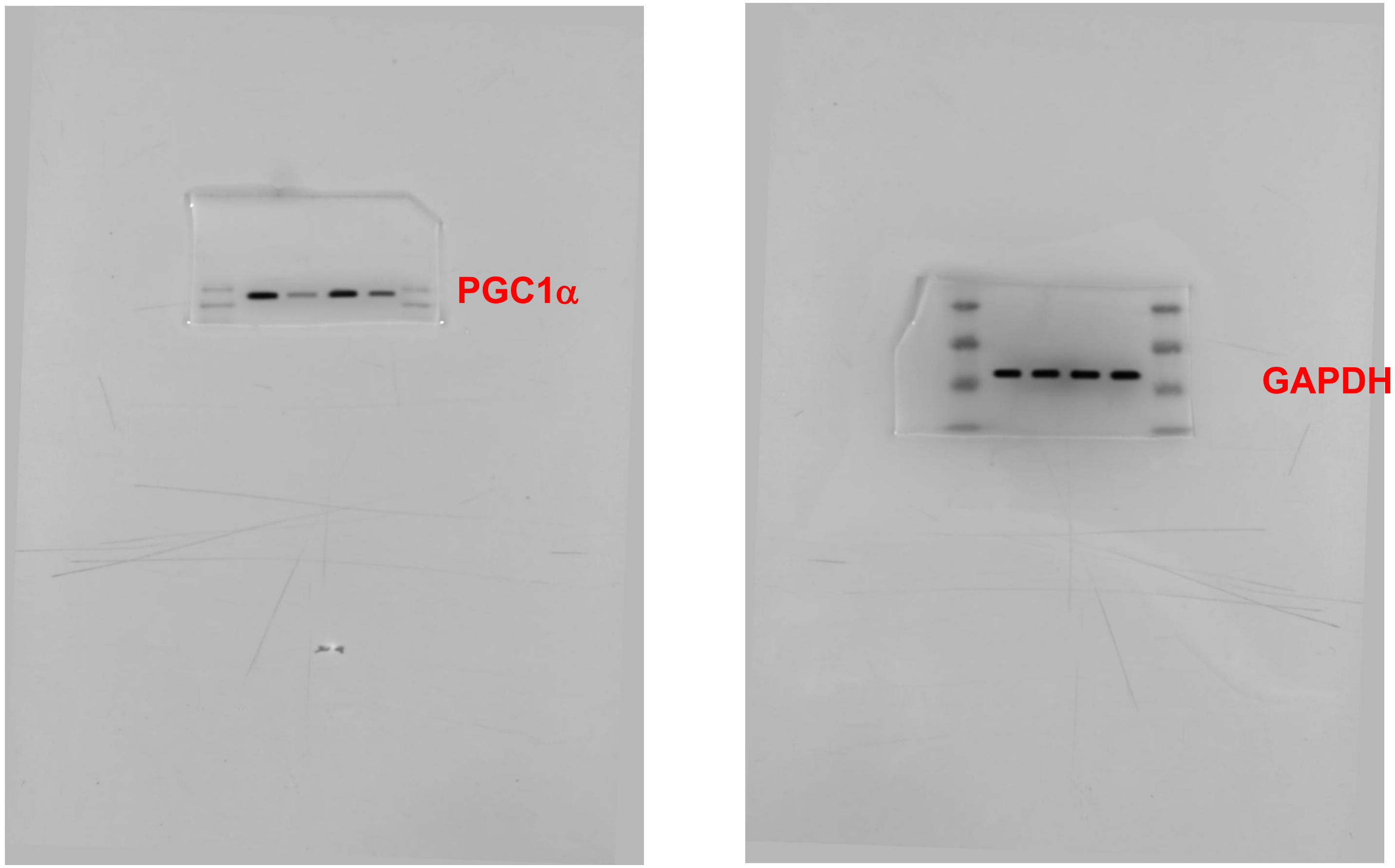

Figure S1

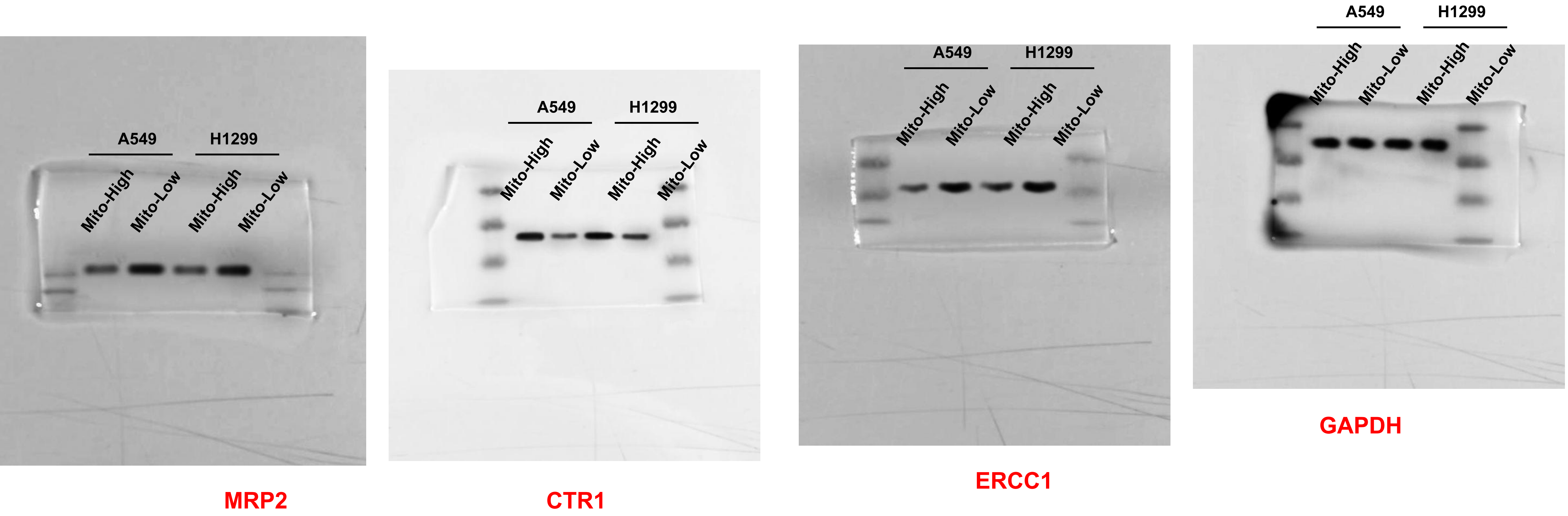
A549
H1299
Mito-High
Mito-Low
Mito-High
Mito-Low
MRP2
CTR1
ERCC1
GAPDH

Figure S6B

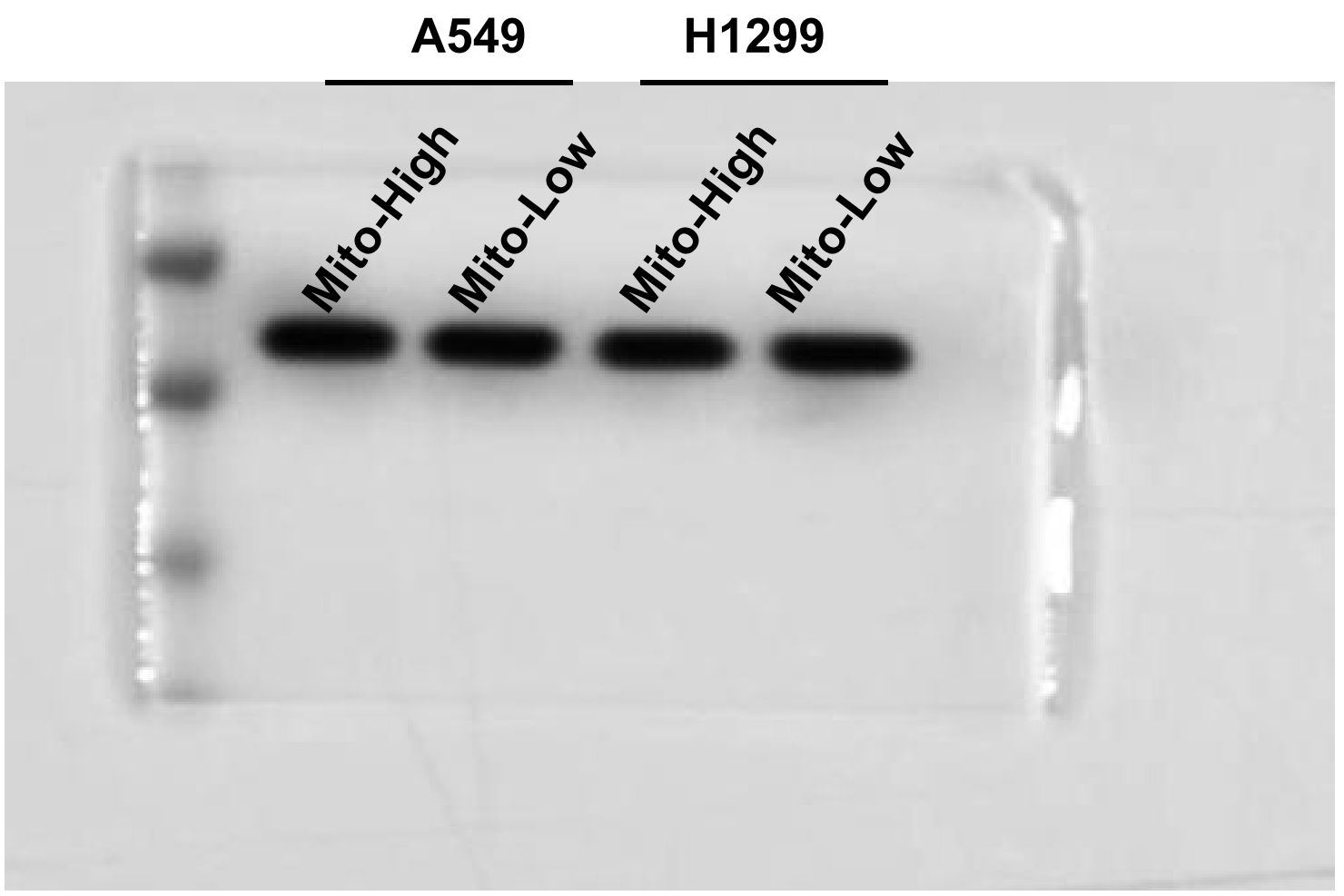

GAPDH

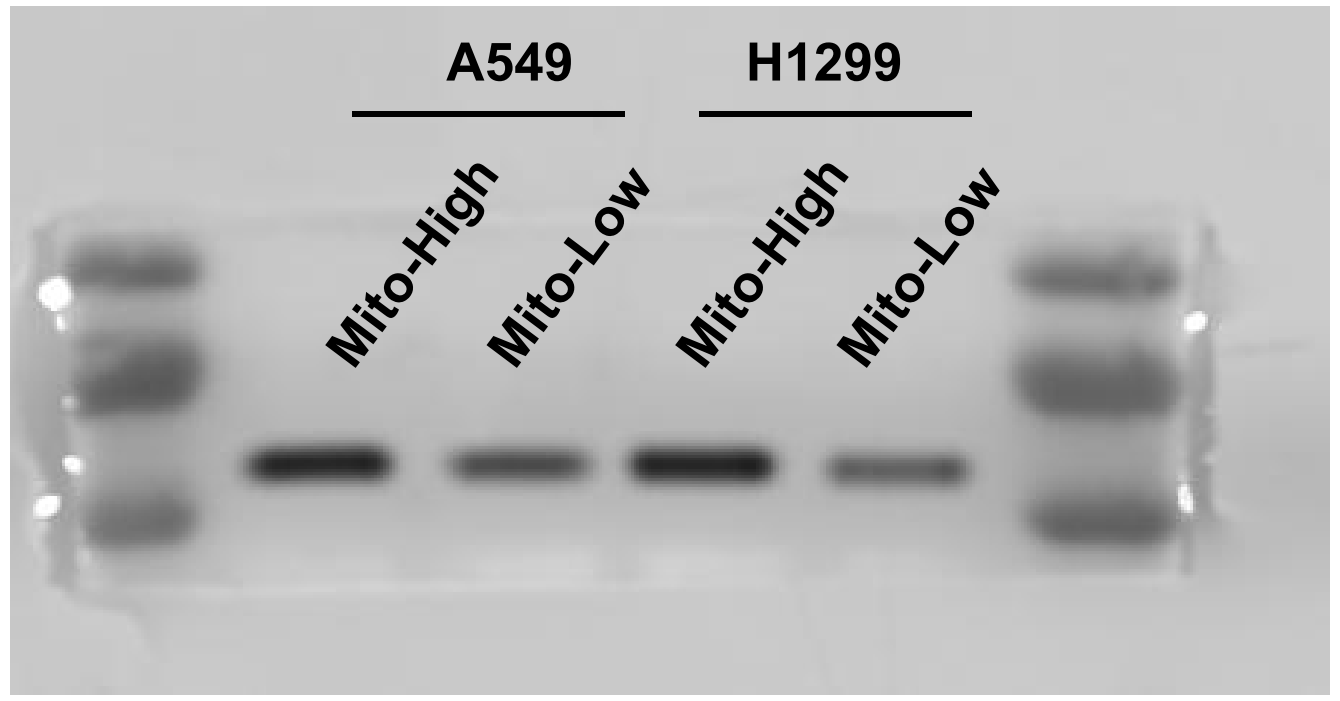

KLF15
