# Supplementary material for: KLF15 activates PGC1α to rewire mitochondrial homeostasis and overcome cisplatin resistance in lung adenocarcinoma
Source: Cell Death Discov. 2026 Jun 27;12:369. doi: 10.1038/s41420-026-03235-3 (PMC13572412; doi:10.1038/s41420-026-03235-3)
Supplement: Supplementary file 3 [file 41420_2026_3235_MOESM1_ESM.docx]

**Figure S1. Validation of classical cisplatin resistance markers in A549/DDP and H1299/DDP cell lines.** Representative Western blot images and corresponding quantitative analysis of MRP2, ERCC1, and CTR1 protein expression levels in parental (A549, H1299) and cisplatin-resistant (A549/DDP, H1299/DDP) lung adenocarcinoma cells. GAPDH was used as the loading control. Data are presented as mean ± SEM. Statistical significance was determined using the two-tailed unpaired Student's t-test. ^***^*P* < 0.001.

**Figure S2. KLF15 overexpression reverses cisplatin resistance in vivo.** TUNEL staining of xenograft sections to quantify apoptosis rates in each treatment group. All data are presented as mean ± SEM from at least three independent experiments. Statistical significance was determined using two-way ANOVA followed by Tukey's post-hoc test. ^***^*P* < 0.001.

**Figure S3.** Statistical analysis of mitochondrial stress test in a Seahorse XF Analyzer to assess basal respiration, maximal respiration, and spare capacity in cisplatin resistant versus parental LUAD cells. All data are presented as mean ± SEM from at least three independent experiments. Statistical significance was determined using one-way ANOVA followed by Tukey's post-hoc test. ^**^*P* < 0.01, ^***^*P* < 0.001.

**Figure S4. Pharmacological inhibition of PGC1α reverses KLF15-induced cisplatin sensitivity.** (A) IC50 analysis of cisplatin sensitivity in KLF15-overexpressing A549 and H1299 cells treated with cisplatin in the presence of vehicle control or the PGC1α pharmacological inhibitor SR-18292 (10 μM). (B) Quantification of colony formation assays in KLF15-overexpressing cells treated with cisplatin ± SR-18292. (C) Apoptosis was evaluated by Annexin V/PI staining and flow cytometry in the indicated cells treated with cisplatin ± SR-18292. Bar graphs show the percentage of apoptotic cells. Data are presented as mean ± SD of three independent experiments. Statistical significance was assessed using a two-tailed unpaired Student’s *t*-test. **P* < 0.05, ***P* < 0.01, ****P* < 0.001.

**Figure S5. KLF15 modulates chemoresistance in lung cancer cells through a PGC1α-dependent mechanism.** **(A)** IC50 analysis of cisplatin sensitivity in A549 and H1299 resistant cells. **(B)** Flow cytometry analysis of apoptosis using Annexin V/PI staining in the indicated A549 and H1299 cell groups following cisplatin treatment. Representative scatter plots (left) and quantification of the percentage of apoptotic cells (right) are shown. Data are presented as mean ± SD of three independent experiments. Statistical significance was determined using two-way ANOVA followed by Tukey's post-hoc test. **P* < 0.05, ***P* < 0.01, ****P* < 0.001.

**Figure S6.** A. qPCR analysis showing lower KLF15 mRNA levels in the Mito low subpopulation. B. Western blot confirming reduced KLF15 protein in Mito low cells. All data are presented as mean ± SEM from at least three independent experiments. Statistical significance was assessed using a two-tailed unpaired Student’s *t*-test. ^**^*P* < 0.01, ^***^*P* < 0.001.
