# Supplementary material for: KLF15 activates PGC1α to rewire mitochondrial homeostasis and overcome cisplatin resistance in lung adenocarcinoma
Source: Cell Death Discov. 2026 Jun 27;12:369. doi: 10.1038/s41420-026-03235-3 (PMC13572412; doi:10.1038/s41420-026-03235-3)
Supplement: Supplementary file 4 [file 41420_2026_3235_MOESM3_ESM.docx]

**Table S1** Sequences of the qRT-PCR primers

| Gene | Primer direction | Sequence (5’ to 3’) |
| --- | --- | --- |
| KLF15 | PCR primer F | TTCTCGTCGCCAAAATGCC |
|  | PCR primer R | CCTGGGACAATAGGAAGTCCAA |
| PGC1α | PCR primer F | TCTGAGTCTGTATGGAGTGACAT |
|  | PCR primer R | CCAAGTCGTTCACATCTAGTTCA |
| PGC1β | PCR primer F | GATGCCAGCGACTTTGACTC |
|  | PCR primer R | ACCCACGTCATCTTCAGGGA |
| NRF1 | PCR primer F | AGTGCTTAGCCCTTGATGAAGA |
|  | PCR primer R | GCTCTGAAGTGACCTCTGGTAT |
| TFAM | PCR primer F | ATGGCGTTTCTCCGAAGCAT |
|  | PCR primer R | TCCGCCCTATAAGCATCTTGA |
| ERRα | PCR primer F | AGGGTTCCTCGGAGACAGAG |
|  | PCR primer R | TCACAGGATGCCACACCATAG |
| GAPDH | PCR primer F | GCACCGTCAAGGCTGAGAAC |
|  | PCR primer R | GCCTTCTCCATGGTGGTGAA |
| PGC1α | CHIP primer F | GCAACTCTTCGGGAGCTGGTA |
|  | CHIP primer R | GACATTTTCTCATTGAGGGAG |
